# MARCKS cooperates with NKAP to activate NF-kB signaling in smoke-related lung cancer

**DOI:** 10.7150/thno.53558

**Published:** 2021-02-19

**Authors:** Jun Liu, Szu-Jung Chen, Ssu-Wei Hsu, Jun Zhang, Ji-Min Li, David C. Yang, Shenwen Gu, Kent E. Pinkerton, Ching-Hsien Chen

**Affiliations:** 1 Department of Internal Medicine, Division of Pulmonary and Critical Care Medicine and Center for Comparative Respiratory Biology and Medicine, University of California Davis, Davis, California, USA; 2 Division of Nephrology, Department of Internal Medicine, University of California Davis, Davis, California, USA; 3 Comprehensive Cancer Center, University of California Davis, Davis, California, USA; 4 Center for Health and the Environment and Department of Pediatrics, University of California Davis, Davis, CA, USA

**Keywords:** MARCKS, NKAP, NF-κB, cigarette smoking, lung cancer

## Abstract

**Rationale:** Cigarette smoking is a major risk factor for lung cancer development and progression; however, the mechanism of how cigarette smoke activates signaling pathways in promoting cancer malignancy remains to be established. Herein, we aimed to determine the contribution of a signaling protein, myristoylated alanine-rich C kinase substrate (MARCKS), in smoke-mediated lung cancer.

**Methods:** We firstly examined the levels of phosphorylated MARCKS (phospho-MARCKS) in smoke-exposed human lung cancer cells and specimens as well as non-human primate airway epithelium. Next, the MARCKS-interactome and its gene networks were identified. We also used genetic and pharmacological approaches to verify the functionality and molecular mechanism of smoke-induced phospho-MARCKS.

**Results:** We observed that MARCKS becomes activated in airway epithelium and lung cancer cells in response to cigarette smoke. Functional proteomics revealed MARCKS protein directly binds to NF-κB-activating protein (NKAP). Following MARCKS phosphorylation at ser159 and ser163, the MARCKS-NKAP interaction was inhibited, leading to the activation of NF-κB signaling. In a screen of two cohorts of lung cancer patients, we confirmed that phospho-MARCKS is positively correlated with phospho-NF-κB (phospho-p65), and poor survival. Surprisingly, smoke-induced phospho-MARCKS upregulated the expression of pro-inflammatory cytokines, epithelial-mesenchymal transition, and stem-like properties. Conversely, targeting of MARCKS phosphorylation with MPS peptide, a specific MARCKS phosphorylation inhibitor, suppressed smoke-mediated NF-κB signaling activity, pro-inflammatory cytokines expression, aggressiveness and stemness of lung cancer cells.

**Conclusion:** Our results suggest that phospho-MARCKS is a novel NF-kB activator in smoke-mediated lung cancer progression and provide a promising molecular model for developing new anticancer strategies.

## Introduction

Lung cancer remains one of leading causes of cancer mortality both in the United States and worldwide 1. Cigarette smoking has long been implicated as a significant factor associated with lung cancer, in which roughly 85% of cases resulting from cigarette smoking 2, 3. A large body of evidence supports the pro-tumorigenic role of cigarette smoking; however, the mechanisms through which cigarette smoking initiates and promotes lung cancer are still an area of active exploration. While non-smoking associated lung cancers have experienced a renaissance of sorts in effective targeted therapies with the advent of therapeutics specific for certain molecular signatures such as epidermal growth factor receptor (EGFR) and ALK inhibitors 4, 5, smoking related lung cancers lack specific druggable targets and remain difficult to treat despite the preponderance of lung cancers related to cigarette smoking. KRAS activation has been implicated as a molecular signature in smoking-related lung cancers; however, specificity issues retard the development of targeted therapies for KRAS and difficulty in generating effective inhibitors and renders the protein “undruggable” 6. Thus, identification and development of suitable molecular targets is of utmost interest to expand the therapeutic options available for smoking-related lung cancer.

A target under active investigation is nuclear factor-kappaB (NF-κB). The NF-κB family is composed of five members: RelA/p65, RelB, c-Rel, p50/p105, and p52/p100 7. Canonical activation of NF-κB signaling is primarily through a heterodimer of p65 and p50 which act as a transcription factor for NF-κB target genes. Under normal conditions, this activation is kept under tight control by inhibitors of kappaBs (IκBs) which bind to NF-κB subunits preventing NF-κB translocation to the nucleus. Upon phosphorylation by IκB kinase (Iκκ), IκB undergoes ubiquitination and subsequent proteasomal degradation, freeing up NF-κB to translocate to the nucleus and regulate target gene transcription 8. NF-κB has been known to be an important regulator of inflammation and to play a role in tumorigenesis in lung cancer 9. Much evidence suggests that NF-κB is an important molecule activated in response to cigarette smoke, promoting tumorigenesis and lung cancer proliferation and survival 10. However, the mechanisms of how NF-κB is activated by cigarette smoke in the context of lung cancer is still not completely defined and an area of current investigation. Beyond the mechanism of activation, NF-κB inhibition is anticipated to be effective, however, specificity issues and potential drawbacks such as immunosuppression and inhibition of anti-tumoral immune response are issues to be resolved before NF-κB targeting can be applied in therapeutic treatment.

Myristoylated alanine-rich C kinase substrate (MARCKS) is a 32kDa membrane associated protein that plays roles in cellular processes such as exocytosis, cell migration, and regulation of the inflammatory response 11. MARCKS is a protein kinase C (PKC) target that shuttles between the membrane and the cytoplasm which is controlled by PKC-dependent phosphorylation and functions in regulating phospholipids at the cell membrane, controlling downstream signaling pathways such as phosphoinositide 3-kinase/protein kinase B (PI3K/AKT)^12^. Prior studies have linked MARCKS activation to tumorigenesis, metastasis and drug resistance suggesting the pro-tumor role of MARCKS in cancer 13, 14. Despite the wealth of evidence of the functionality of MARCKS in various cancers, it is unknown if MARCKS' contribution to cancer progression can be activated by cigarette smoke. In this study, we investigated the role of MARCKS in response to cigarette smoke and its participation in downstream signaling to promote lung cancer malignancy.

## Materials and Methods

### Reagents and antibodies

All reagents and antibodies used in this study are described in the Supplementary Methods in the online supplement.

### Cell culture and transfection

The human lung cancer cell lines, CL1-0 and CL1-5 were established as previously described 15. The lung cancer cell lines H292 and A549 were purchased from the American Type Culture Collection (ATCC) (Manassas, VA). Cancer cell lines were cultured in RPMI-1640 medium with 10% fetal bovine serum (FBS) and 1% penicillin-streptomycin at 37 °C in a humidified atmosphere of 5% CO_2_. The human HBE1 cell line was a gift from JR Yankaskas, University of North Carolina, and were grown in Clonetics BEGM medium (Cambrex Lonza, East Rutherford, New Jersey) with all hormones/growth factors included in the package, except the retinoic acid. Detailed experimental procedures for establishment of cell culture and siRNA transfections are described in the Supplementary Methods in the online supplement.

### Exposure of cultured cells to cigarette smoke extract

Cultures of cells were exposed to cigarette smoke extract (CSE) using a protocol similar to that previously described 16. Detailed experimental procedure for generation of CSE is described in the Supplementary Methods in the online supplement.

### Non-human primates of cigarette smoke exposure

The paraffin-embedded specimens of rhesus macaques exposed to filtered air (FA; control) and environmental tobacco smoke (ETS) were kindly provided from Dr. Kent E. Pinkerton (Centre for Health and the Environment, UC Davis). Detailed procedures for animal model of smoke exposure are described in the Supplementary Methods in the online supplement.

### Patient tumor specimens and immunohistochemical staining

Lung tumor specimens were obtained from patients with histologically confirmed lung cancer who underwent surgical resection at the UC Davis Medical Center. None of the patients had received pre-operative adjuvant chemotherapy or radiation therapy. This investigation was approved by the Institutional Review Board of the UC Davis Health System. Written informed consent was obtained from all patients. Formalin-fixed and paraffin-embedded specimens were used, and immunohistochemical staining was performed for phospho-MARCKS and phospho-p65 levels as described previously 13, 15, 17. Detailed information on staining and scoring is found in the Supplementary Methods in the online supplement.

### Liquid chromatography-tandem mass spectrometry (LC-MS/MS) assays

The precipitated proteins from 5×10^6^ cells using anti-V5 antibody were collected and submitted to the UC Davis Proteomics Core Facility (Davis, CA) for LC-MS/MS analysis. Proteins were identified and selected according to their involvement in crucial cellular functions and signaling pathways.

### Quantitative real-time PCR and RNA sequencing analysis

Primers used for quantitative real-time PCR is shown in Table S1. The detailed procedures for RNA extractions, quantitative real-time PCR and transcriptome profiling analysis are described in the and Supplementary Methods in the online supplement.

### Cell invasion, scratch wound-healing and oncosphere-forming assays

An *in vitro* cell invasion assay was performed as previously described 13, 15, 18 using Transwell chambers (8-μm pore size; Costar, Cambridge, MA). Briefly, 2×10^4^ cells were seeded on top of the polycarbonate filters coated with Matrigel (Becton Dickinson, Franklin Lakes, NJ), and 0.5 mL of growth medium was added to both the upper and lower wells. After incubation for 20 hours, filters were swabbed with a cotton swab, fixed with methanol, and then stained with Giemsa solution (MilliporeSigma, Burlington, MA). The cells attached to the lower surface of the filter were counted under a light microscope (10X magnification). For a wound-healing assay, cells were seeded to six-well tissue culture dishes and grown to confluence. Each confluent monolayer was then wounded linearly using a pipette tip and washed three times with PBS. Thereafter, cell morphology and migration were observed and photographed at 0 h and 12 h. The number of cells migrating into the cell-free zone was acquired under a light microscope. For oncosphere formation assay, cells were placed in ultra-low adherent plate after single cell suspension. Cells grown in sphere formation medium containing B27, 20 ng/mL epidermal growth factor, 10 ng/mL basic fibroblast growth factor, 5 μg/mL insulin, 0.4% bovine serum albumin for two weeks. The number and size of spheres were counted under a microscope.

### Western blot, immunoprecipitation, immunofluorescent staining, flow cytometry, and* in vivo* tail vein metastasis assays

Detailed procedures for Western blot analyses, immunoprecipitation, immunofluorescent staining, flow cytometry, and *in vivo* tail vein metastasis assays are described in the Supplementary Methods in the online supplement.

### Statistical analysis

Data analysis procedures are described in the Supplementary Methods in the online supplement.

## Results

### MARCKS becomes activated in response to cigarette smoke

**Figure 1:**
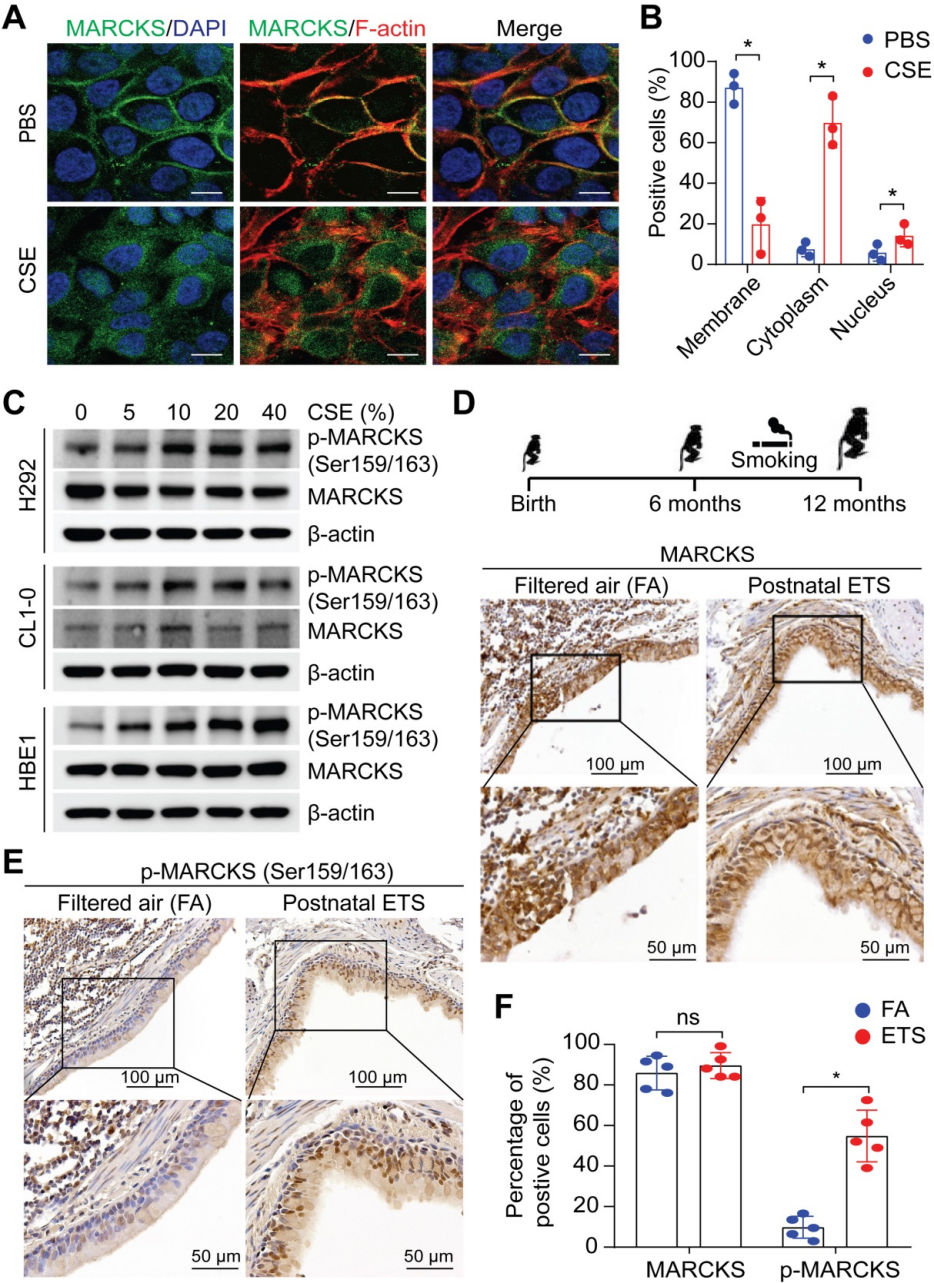
** MARCKS is activated/phosphorylated in response to tobacco smoke. (A)** Representative images of MARCKS localization in H292 cells treated with or without 10% cigarette smoke extracts (CSE) for 24 hours, scale bar = 20μm. **(B)** Quantification of the subcellular localization of MARCKS in response to CSE. Data are from three independent repeated assays; *, *p* < 0.05. **(C)** Western blot analysis of total MARCKS and phospho-MARCKS expression in H292, CL1-0, and HBE1 cells exposed to the indicated doses of CSE for 24 hours. β-actin was used as the loading control. PANEL **(D-F)**: Lung tissues from rhesus macaques (*n* = 5) exposed to filtered air (FA) or environmental tobacco smoke (ETS) for 6 months were collected and then subjected to IHC staining for MARCKS and phospho-MARCKS expression, respectively. Representative IHC staining images and quantification of bronchial epithelial cells positive for MARCKS **(D, F)** and phospho-MARCKS **(E, F)** respectively. ns: no significance; *, *p* < 0.05, Student's *t*-test.

Upregulation of MARCKS, particularly phosphorylated MARCKS (phospho-MARCKS) at the phosphorylation site domain (PSD), has recently emerged as both a biomarker and therapeutic target for lung cancer 11, 13, 15, 17, 19, 20, a malignancy etiologically associated with cigarette smoking 2. Given that cigarette smoking is a well-known driver of malignant phenotypes in lung cancer 21, we first determined if exposure to smoke regulates MARCKS protein. Minimally-invasive human lung cancer cells, H292 cells, were utilized for the exposure of cigarette smoke extracts (CSE) due to its low expression of phospho-MARCKS 15. Immunofluorescence staining data showed that the detachment of MARCKS protein from the cell membrane occurred in most cells exposed to CSE, suggesting that smoke exposure promotes MARCKS phosphorylation and activation (Figure 1A). In addition to cytoplasmic distribution, we observed a small proportion of MARCKS protein localized in the nucleus in response to smoke. An increase of MARCKS translocation to the cell cytoplasm and nucleus was noted in CSE-treated cells compared to that in cells incubated with PBS (Figure 1B). Western blots also confirmed that smoke exposure increased MARCKS phosphorylation in less-invasive human lung cancer cell lines (H292 and CL1-0, epithelial-like) and lung epithelial cells (HBE1) in a dose-dependent manner (Figure 1C). To further confirm the above observations that cigarette smoke facilitates increased phospho-MARCKS levels *in vivo*, paraffin-embedded lung tissue specimens of rhesus macaques exposed to filtered air (FA; control) and environmental tobacco smoke (ETS) 22 were subjected to immunohistochemistry (IHC) staining using both anti-phospho-MARCKS and anti-MARCKS antibodies. Despite no enhancement of IHC staining signal for total MARCKS expression (Figure 1D, 1F), a significant higher intensity of staining and more stained bronchial epithelial cells were observed in smoke-exposed lung tissues than those in the FA controls (Figure 1E-F). These data strongly suggest that cigarette smoking promotes MARCKS phosphorylation to activate MARCKS activity in the lung.

### MARCKS interacts with NKAP and is involved in NF-κB signaling

**Figure 2:**
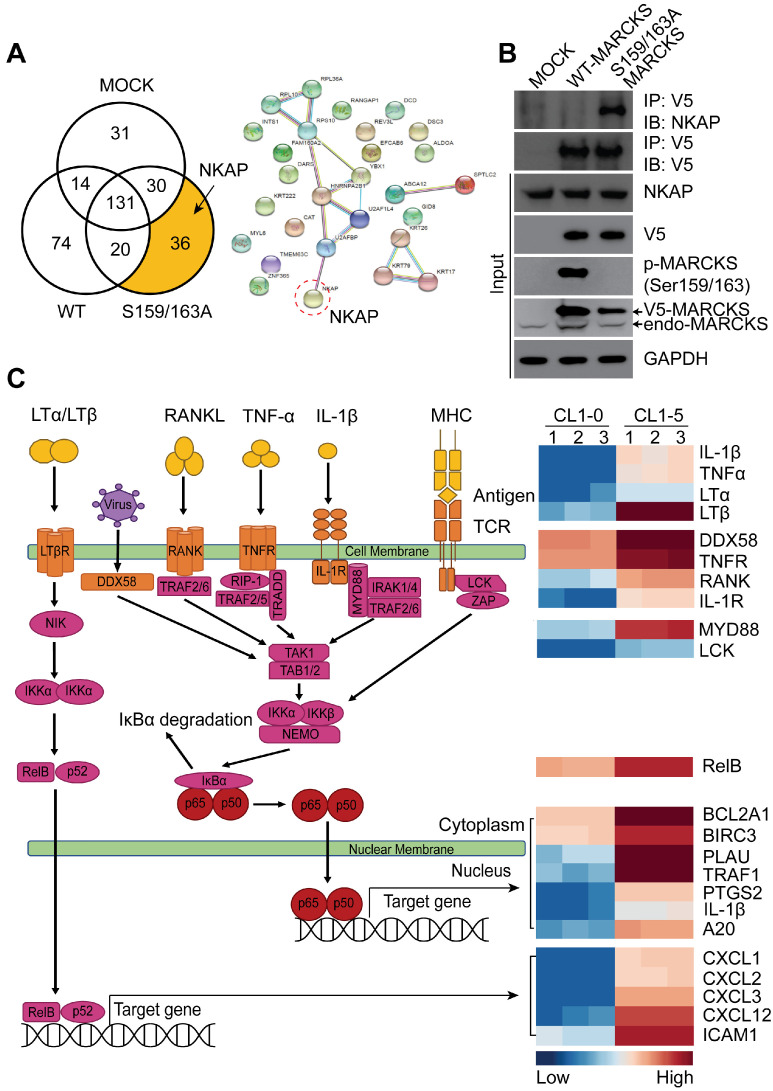
** MARCKS interacts with NKAP and participates in NF-κB signaling. (A)** Functional proteomic analysis of MARCKS-interacting proteins identified by mass spectrometry. Left, number of proteins bound to phosphorylated or unphosphorylated MARCKS protein. Right, the potential unphosphorylated MARCKS-interacting proteins. **(B)** Co-immunoprecipitation analysis of the association between MARCKS and NKAP. V5-tagged proteins was precipitated by using anti-V-5 antibody in CL1-0 cells transfected with V5-tagged wild-type MARCKS, V5-tagged S159/163A MARCKS or mock control (empty vector only). **(C)** Heatmap analysis from RNA-seq data showing the differentially expressed genes associated with NF-κB signaling in highly phospho-MARCKS-expressing CL1-5 cells as compared to CL1-0 cells with low phospho-MARCKS expression. Red shades indicate higher intensity and blue shades represent lower intensity. Data are significant changes as determined by a p-value threshold of less than 0.05.

Since most signaling proteins, including MARCKS protein, interact with other proteins for driving biological processes 11, we investigated whether MARCKS forms oncogenic complexes for executing its pro-tumor activities. To identify the molecular complexes formed by unphosphorylated and phosphorylated MARCKS, V5-tagged wild-type (phosphorylation-abundant) and S159/163A mutant (phosphorylation-defective) of MARCKS expression constructs were respectively transfected into low MARCKS-expressing CL1-0 cells, as we reported previously 13, 15. After 48 hours of transient transfection, V5 alone (Mock control), V5-tagged wild-type MARCKS and S159/163A mutant MARCKS proteins were precipitated and subjected to liquid chromatography-tandem mass spectrometry (LC-MS/MS). LC-MS/MS successfully identified 336 proteins in total. Among the identified hits, 74 proteins were found to specifically interact with phosphorylated MARCKS, and 36 proteins were shown to specifically bind to unphosphorylated MARCKS (Figure 2A, left). The NF-κB activating protein (NKAP), a protein identified in our analysis to interact with unphosphorylated MARCKS, drew our attention owing to its role in directly activating NF-κB signaling, a smoke-related signaling pathway associated with cancer malignancy 10, 23, 24 (Figure 2A, right). Co-immunoprecipitation assays confirmed a strong binding between phosphorylation-defective S159/163A-MARCKS and NKAP in CL1-0 cells but this interaction did not occur in phosphorylation-abundant MARCKS (Figure 2B). An association between endogenous MARCKS and NKAP was also validated in MARCKS-proficient CL1-5 cells (Figure S1A). However, this interaction did not occur between phospho-MARCKS and NKAP, implying that MARCKS is disassociated from NKAP after its PSD motif is phosphorylated.

Given that PKC is a major kinase for MARCKS phosphorylation at the PSD, we next determined the specific PKC isoforms involved in smoke-enhanced phospho-MARCKS. Cells were preincubated with various isoform-specific PKC inhibitors for 30 minutes followed by co-incubation with CSE. We found that two of the inhibitors, alpha-isoform-selective (Gö6976) and delta-isoform-selective (Rottlerin) PKC inhibitors, reduced phospho-MARCKS abundance in smoke-exposed cells (Figure S1B). Interestingly, an interaction between MARCKS and NKAP was downregulated in response to smoke exposure, whereas treatment with an alpha-isoform-selective or a delta-isoform-selective PKC inhibitor to suppress phospho-MARCKS abundance could restore MARCKS binding to NKAP in CSE-exposed cells (Figure S1C). Consistent with the co-immunoprecipitation data, immunofluorescent staining has shown an increase of co-localization of MARCKS and NKAP in CSE-exposed cells after treatment with the two PKC inhibitors (Figure S1D). These above results suggest that a unique interaction between MARCKS and NKAP depends on the phosphorylation status of MARCKS.

Highly metastatic CL1-5 cells, a stable sub-line derived from non-metastatic CL1-0 parent line, was previously reported to exhibit higher invasive capability and phospho-MARCKS abundance as compared to CL1-0 cells 13, 15. Through a comparison of transcriptome profiling between CL1-0 and CL1-5 cells using RNA sequencing (SRA accession number: PRJNA689089), followed by analysis with the DAVID pathway analysis tool (https://david.ncifcrf.gov), we noticed significant upregulation of the NF-κB pathway in CL1-5 cells (Figure S2A). Figure 2C shows a heatmap of the top differentially expressed genes (DEGs) associated with the NF-κB pathway. We next evaluated whether there was an association of these DEGs with overall survival in lung cancer patients from The Cancer Genome Atlas (TCGA). Data from Kaplan-Meier plotter analysis of the TCGA dataset for lung cancer with smoke history (n = 820) showed that the expression of MARCKS and NF-κB signaling gene signature, including TNFR, RELA, BCL2A1, CXCL1, CXCL3, LTA, RELB and TNF-alpha, was associated with poor survival of lung cancer patients (Figure S3A). In a screen of another cohort of lung cancer patients with smoking history (n = 151), downregulated expression of the inhibitory element of NF-κB signaling NFKBIA, which encodes the NF-κB inhibitory protein IκBα, was seen in lung cancer tissues from smokers and positively correlated with poor outcome (Figure S3B-C). We also computed the correlation between MARCKS gene and NF-κB signaling gene signature in lung cancer specimens from The Cancer Genome Atlas (TCGA) and normal lung tissues from the Genotype-Tissue Expression (GTEx) databases. Data from Gene Expression Profiling Interactive Analysis (GEPIA) 25 analysis confirmed a significantly positive correlation of MARCKS expression with TNFR, RELA, BCL2A1, CXCL1, RELB or TNF-alpha in lung cancer samples but not in normal lung tissues (Figure S4A).

### Co-upregulation of phospho-MARCKS and phospho-NF-κB p65 in lung cancer

**Figure 3:**
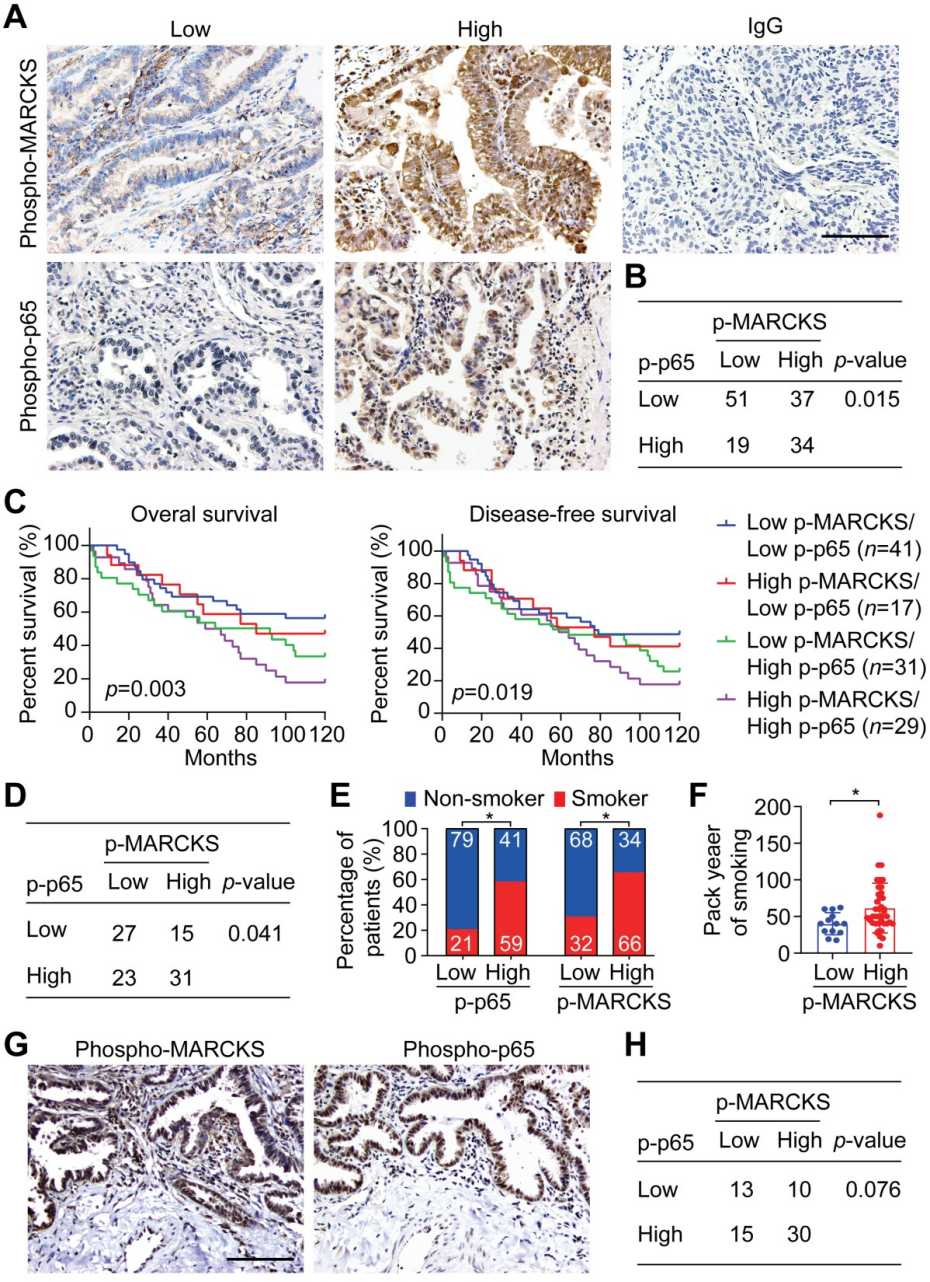
** Co-expression of phospho-MARCKS and phospho-p65 occurred in lung cancer specimens of smokers and correlated with poor outcome. (A)** Representative images of immunohistochemical staining using anti phospho-MARCKS and phospho-p65 in lung cancer specimens. Scale bar = 100 μm. **(B)** Correlation of phospho-MARCKS and phospho-p65 in a cohort of 141 lung patients. **(C)** Overall survival and diseases-free survival of lung cancer patients were analyzed by Kaplan-Meier plot and two-sided log-rank test (n *=* 118). **(D)** Correlation of phospho-MARCKS and phospho-p65 expression in another cohort of 96 lung cancer patients. **(E)** Percentage of 96 patients with high- and low-expression of phospho-MARCKS and phospho-p65 according to their smoking status. **(F)** Higher smoking pack-year was found in lung cancer patients with high phospho-MARCKS level (n = 52); *, *p* < 0.05. **(G)** Co-expression of phospho-MARCKS and phospho-p65 in lung cancer specimens from smokers. Scale bar = 100 μm. **(H)** An association between phospho-MARCKS and phospho-p65 in 68 lung cancer specimens with smoke history.

In light of the above observations that activation of NF-κB signaling is associated with lung cancer malignancy, we further confirmed the phosphorylation levels of MARCKS and p65 (S536), an indicator of the activated form of NF-κB, in lung cancer specimens by performing IHC staining. Patient samples were further grouped into low- and high-expression categories (Figure 3A; n = 141) and their clinical characteristics were summarized in Tables S2, S3 and S4. Our analyses revealed a positive association between phospho-MARCKS and phospho-p65 in these lung cancer specimens (Figure 3B; *p* = 0.015, Fisher's exact test). Surprisingly, the patients with both high levels of phospho-MARCKS and phospho-p65 had a significantly shorter overall survival as compared to those patients with low phospho-MARCKS and phospho-p65 (Figure 3C, left; *p* = 0.003, log-rank test). Additionally, Kaplan-Meier survival analysis followed by the log-rank test demonstrates the worst disease-free survival in the high phospho-MARCKS and phospho-p65 group (Figure 3C, right;* p* = 0.019), suggesting co-expression of phospho-MARCKS and phospho-p65 is likely of more importance in patients with poor prognosis.

To examine the clinical relevance of the co-expression of phospho-MARCKS and phospho-p65 in smoke-related lung cancer, we then validated both phospho-MARCKS and phospho-p65 signal abundance in another cohort of lung cancer patients (n = 96) with smoking history (Tables S5, S6 and S7). As expected, patients with cigarette use displayed the worst overall survival and disease-free survival (Figure S5A-B). Consistently, a positive correlation between phospho-MARCKS and phospho-p65 was established (Figure 3D; *p* = 0.041, Fisher's exact test). High phospho-p65 expression in samples was significantly greater in patients from smokers compared to patients from non-smokers, at a rate of 59% (n = 40/68) versus 41% (n = 28/68), respectively (Figure 3E, left). Likewise, higher levels of phospho-MARCKS were detected in 66% (n = 45/68) of lung cancer specimens from smokers (Figure 3E, right). We also noticed a higher smoking pack-year in lung cancer patients with high phospho-MARCKS (Figure 3F). Analysis of co-expressing phospho-MARCKS and phospho-p65 abundance was achieved by using two sequential tissue slices of lung cancer samples from smokers to ensure that images are properly coregistered. Figure 3G shows overlapping staining between phospho-MARCKS and phospho-p65 abundance. Simultaneously, co-upregulation of phospho-MARCKS and phospho-p65 was seen in smoke-related lung cancer (Figure 3H).

### Phosphorylation status of MARCKS regulates NF-κB signalling activity

**Figure 4:**
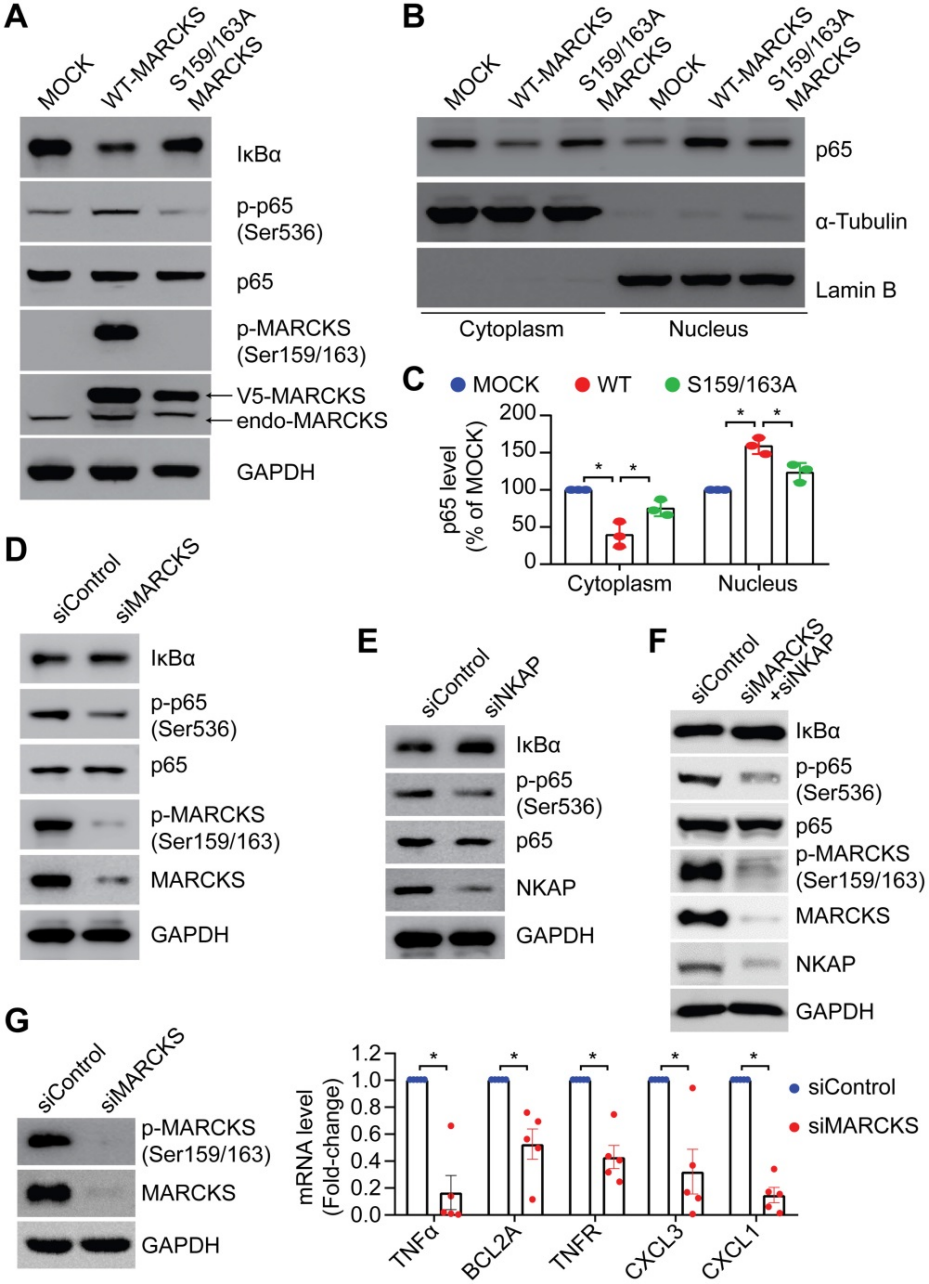
** MARCKS modulates NF-κB singling through NKAP expression. PANEL A-C**: Effects of ectopic V5-tagged wild-type or PSD-mutated (S159/163A) MARCKS expression on MARCKS phosphorylation, NF-κB signaling activity** (A)** and p65 localization** (B)** in CL1-0 cells. **(C)** Quantification of western blots for p65 localization in the cytoplasm and nucleus of CL1-0 cells transfected with V5-tagged wild-type MARCKS, V5-tagged S159/163A MARCKS or mock control. Data from three independent experiments are represented as mean ± SD; *, *p* < 0.05. **(D)** Effects of MARCKS siRNA silencing on NF-κB signaling activity and MARCKS phosphorylation in CL1-5 cells. **(E)** Western blot analysis of NF-κB signaling activity upon siRNA knockdown of NKAP expression in CL1-5 cells.** (F)** Effects of dual knockdown of MARCKS and NKAP on NF-κB signaling activity in CL1-5 cells. **(G)** Expression of NF-κB target genes in response to MARCKS knockdown. Left, MARCKS protein and its phosphorylation in control siRNA and MARCKS-knockdown CL1-5 cells were determined after 72 hours of siRNA transfection. Right, control siRNA and MARCKS-knockdown CL1-5 cells were harvested for RNA isolation and the level of gene expression was quantified with real-time RT-qPCR and normalized with the TBP level. Data expressed as mean ± SEM (*n* = 5; *, *p* < 0.05).

To elucidate whether phospho-MARCKS regulates NF-κB signaling activation, we carried out ectopic expression of V5-tagged wild type and PSD-mutated (S159/163A) MARCKS in low MARCKS-expressing CL1-0 cells. As shown in Figure 4A, cells with overexpression of V5-tagged wild type MARCKS exhibited an elevated level of phospho-p65 and IκBα degradation, whereas no significant upregulation of phospho-p65 and IκBα degradation were noted in cells transfected with PSD-mutated MARCKS. Since Serine 536 phosphorylation affects p65 nuclear import 7, we further assessed subcellular localization of p65 in the above cells. Western blots confirmed concomitantly increased nuclear fractions and decreased cytosolic fractions of p65 proteins in cells ectopically expressing V5-tagged wild type MARCKS, but not in cells transfected with PSD-mutated MARCKS (Figure 4B-C). Conversely, knockdown of MARCKS expression in CL1-5 cells using MARCKS-specific siRNAs reduced phospho-p65 expression and increased IκBα level (Figure 4D). Furthermore, we utilized NKAP-specific siRNAs to determine if activation of NF-κB signaling in MARCKS-expressing CL1-5 cells is mediated by NKAP. After 72 hours of knockdown of NKAP expression, phospho-p65 abundance was decreased and IκBα level was increased in CL1-5 cells (Figure 4E). Dual knockdown of MARCKS and NKAP was also demonstrated to diminish NF-κB signaling activity (Figure 4F). To determine a causal effect of MARCKS on major target genes of the NF-κB signaling pathways, we examined expression of these target genes indicated in Figure S4 and found that MARCKS-silenced cells display significantly lower mRNA expression of NF-κB target genes as compared to cells receiving control siRNA (Figure 4G). Altogether, these results support the notion that phosphorylated MARCKS promotes NKAP-mediated NF-κB activation and downstream transcriptional targets.

### Smoke-induced phospho-MARCKS promotes NF-κB activation

**Figure 5:**
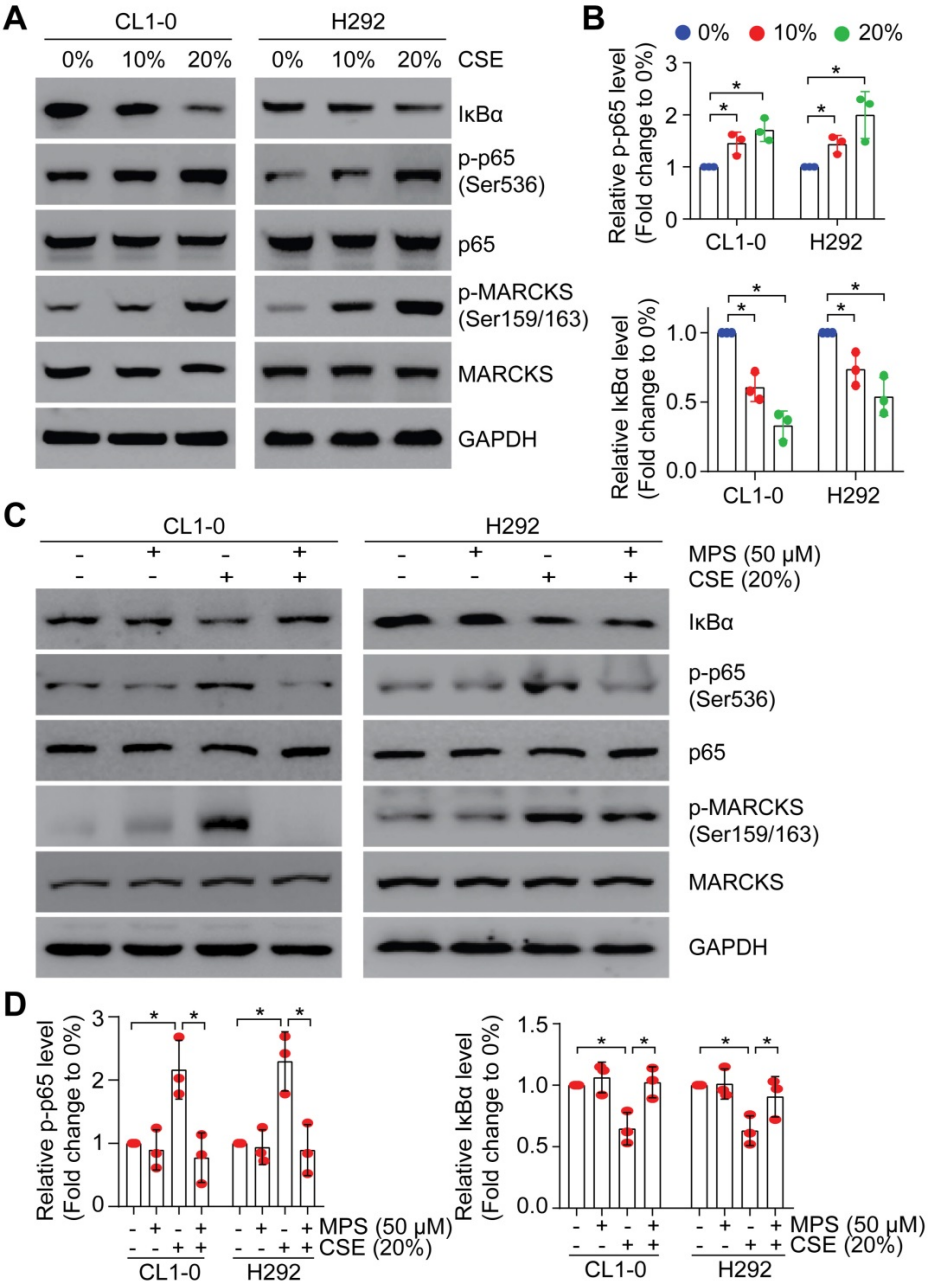
** Smoke exposure induces MARCKS-mediated NF-κB activation.** PANEL **A**-**B**: CSE exposure induced activation of MARCKS and NF-κB in both CL1-0 and H292 cells. Cells were incubated with various doses of CSE as indicated. After 24 hours, cells were collected and subjected to Western blot analysis **(A)**. **(B)** The mean results for densitometric scans of three blots from multiple experiments were expressed as fold change relative to untreated cells. *, *p* < 0.05. PANEL **C**-**D**: MPS peptide, a MARCKS inhibitor, inhibited CSE-induced MARCKS phosphorylation of lung cancer cells. CL1-0 (left) and H292 (right) cells were co-treated with 50 µM MPS peptide and 20% CSE for 24 hours and subjected to immunoblot analysis **(C)**.** (D)** The mean results for densitometric scans of three blots from multiple experiments were expressed as fold change relative to untreated cells. *, *p* < 0.05.

Since smoke exposure drives MARCKS activation/phosphorylation and activated MARCKS increases NF-κB signaling activity, we asked whether NF-κB signaling is activated upon smoke exposure. To examine the effect of smoke exposure on NF-κB signaling, we performed a dose-course analysis of CL1-0 and H292 cells undergoing CSE treatment. We showed that phospho-p65 abundance was increased and IκBα level was decreased in a concentration-dependent manner (Figure 5A), concomitant with induced MARCKS activation. Notably, treatment with 20% CSE resulted in an approximately 1.9-fold increase of phospho-p65 expression and 2.3-fold decease of IκBα level (Figure 5B). We previously identified a small peptide, the MPS peptide, which targets the MARCKS PSD and inhibits MARCKS-mediated functions with no cytotoxic effect on normal human epithelial cells 13, 18, 26, 27. Through treatment with 50 μM MPS peptide in lung cancer cells exposed to 20% CSE, we confirmed that MPS peptide had an inhibitory effect on smoke-enhanced MARCKS phosphorylation in both CL1-0 and H292 cells (Figure 5C). We also noticed that smoke-induced phospho-p65 upregulation and IκBα degradation were suppressed in MPS-treated cells (Figure 5C-D), suggesting that high phospho-MARCKS abundance caused by smoke exposure contributes to the activation of NF-κB signaling in lung cancer cells.

### Pharmacologic inhibition of phospho-MARCKS impairs smoke-mediated cancer malignancy

**Figure 6:**
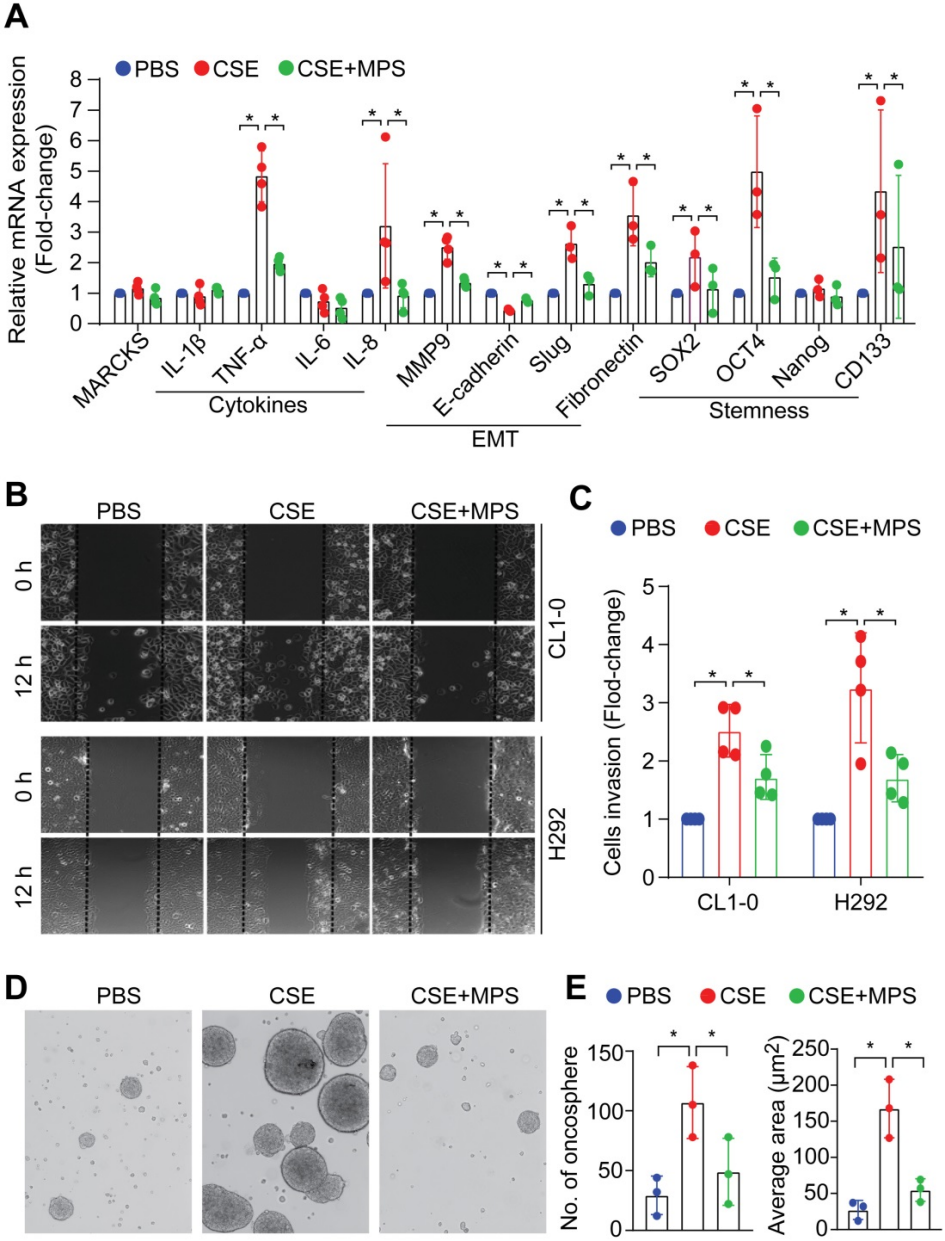
** MARCKS inhibition impairs smoke-induced aggressiveness and stemness of lung cancer cells. (A)** mRNA expression of cytokines, epithelial to mesenchymal transition markers and stemness-related genes in H292 cells as measured by RT-qPCR. Data expressed as mean ± SEM (*n* = 3; *, *p* < 0.05). **(B)** A scratch/wound healing assay for evaluating cell migration. Cells were pretreated with 50 µM MPS peptide for 16 hours and then co-treated with or without 20% CSE. After 24 hours of co-treatment, these cells were scratched and wound healing repair was microscopically examined at 12 h after the scratch. *, *p* < 0.05 (*n* = 3). **(C)** The inhibitory effect of MPS peptide on smoke-mediated cell invasiveness was determined by Matrigel transwell invasion assays. Cells were pretreated with 50 µM MPS peptide for 16 h and then co-treated with or without 20% CSE. After 24 h of co-treatment, these cells were subjected to Matrigel transwell invasion assays. Data expressed as mean ± SD (*n* = 3); *, *p* < 0.05. PANEL **D**-**E**: Sphere-forming assays for evaluating the inhibitory effect of the MPS peptide on smoke-mediated stemness. **(D)** Phase contrast photomicrographs of oncospheres in non-adherent 3-D culture system: PBS alone (left), 10% CSE alone (middle), and 10% CSE plus 50 µM MPS (right) for 14 days. **(E)** Quantification of the number (left) and size (right) of oncospheres. Data expressed as mean ± SD (*n* = 3); *, *p* < 0.05.

Cancer malignancy is characterized by epithelial-mesenchymal transition (EMT), stem-like properties, and elevated expression of pro-inflammatory cytokines that are regulated by activation of NF-κB signaling 28, 29. To determine the phospho-MARCKS functions in smoke-mediated lung cancer malignancy, MPS peptide at a dose of 50 μM was used in combination with 20% CSE to treat cells for 48 h. Analysis of mRNA expression demonstrated that cytokine-related genes including TNF-alpha, IL-8, and EMT markers including Slug and fibronectin, as well as stemness-associated markers including Oct4, Sox2, and CD133 were significantly upregulated in smoke-exposed cells, while no upregulation of the above markers was seen with concurrent MPS peptide treatment (Figure 6A). To characterize the effects of smoke-induced MARCKS phosphorylation on cell phenotypes relevant to cancer cell aggressiveness, we carried out both scratch/wound healing and Matrigel transwell invasion assays. After CSE treatment, a 2.5-fold and 3.3-fold increase in cell migration capability was observed in CL1-0 and H292 cells respectively, whereas MARCKS inhibition by MPS treatment suppressed smoke-enhanced cell migration (Figure 6B and Figure S5C). A similar suppression on cell invasive ability in Matrigel-coated transwell was recapitulated in MPS-treated cells (Figure 6C).

Next, we confirmed the fact that long-term exposure to smoke potentiates cancer stemness 30-34. Serum-free medium and non-adherent culture conditions were used to culture and enrich the cancer stem-like population from CL1-0 cells, which were originally cultured under an adhering culture condition. With non-adherent serum-free culture conditions for 14 days of exposure to PBS or 10% CSE, smoke-incubated cells exhibited higher oncosphere-forming ability and elevation of various stemness-associated transcriptional factors (Figure S5D). Pharmacologically, we treated smoke-enriched oncospheres derived from H292 cells with MPS peptide to target the MARCKS PSD. Figure 6D-E show inhibitory effects of the MPS peptide on the number and size of oncospheres. Such inhibition of cancer stemness by MPS peptide may be attributed to the suppression of smoke-induced MARCKS phosphorylation. Given that KRAS mutations appear to be an early event in smoke-related lung cancer 6, a KRAS-mutated human lung cancer cell line, A549 (G12S), was utilized to test the anti-stemness effect of MPS peptide *in vivo*. Flow cytometry analysis has confirmed higher levels of stemness-associated markers in A549 oncospheres as compared to A549 cells in adherent conditions (Figure S6A-B). In a tail vein metastasis* in vivo* model, mice injected with A549 oncospheres developed more metastatic nodules than those mice received a tail vein injection of adherent A549 cells (n = 7 mice/group) (Figure S6C). However, a significant decrease of metastatic nodules was observed in the lungs and livers of A549 oncospheres-bearing mice treated with 28mg/kg MPS peptide as compared to A549 oncospheres-bearing mice given treatment with PBS (Figure S6C). These data convincingly demonstrate that pharmacologic inhibition of phospho-MARCKS by MPS peptide mitigates smoke-mediated cancer malignancy *in vitro* as well as lung cancer metastasis* in vivo*.

## Discussion

Despite the advances seen in lung cancer therapy during the recent decades, smoking-associated lung cancer remains an intractable disease with few targeted therapies 3. EGFR and ALK tyrosine kinase inhibitors have demonstrated marked activity in lung cancers harboring driver mutations in these genes; however, these mutations are not driving features of smoking-associated lung cancer 35. KRAS activation and mutations have been identified as a tumor-promoting signal in this type of lung cancer but remains undruggable due to difficulties in developing inhibitors for this protein 6, 36. In addition, there exist few markers for predicting patient outcomes and cancer behaviors for smoking-associated lung cancer. Smoking status and higher pack year account for a worse prognosis in patients, indicating that there are features of cigarette smoking which dispose these patients to more aggressive cancer phenotypes 37. Thus, there is a need to identify smoke-associated molecular markers and targets present in lung cancer. Herein, we have found that MARCKS is a smoke-associated marker and a novel smoke responsive molecule.

We have previously identified MARCKS as a key signaling protein upregulated in lung cancer driving tumor cell activities and progression 13, 17. Recent reports have also indicated that various insults can activate MARCKS 38, 39, but the role of MARCKS in response to cigarette smoke has not yet been elucidated. MARCKS is typically localized to the membrane in its unphosphorylated state and upon activation/phosphorylation, MARCKS readily dissociates from the membrane and enters the cytosol 11, 12. We have found MARCKS activity (phospho-MARCKS) was dose-dependently elevated in response to CSE as evidenced by both increases of protein phosphorylation as well as localization to the cytoplasm. Elevation of phospho-MARCKS was also confirmed in an animal model of environmental cigarette smoke exposure, suggesting that MARCKS is indeed a smoke-responsive molecule. Additionally, higher phospho-MARCKS levels found in lung cancer patient tissue and were associated with smoking status as well as higher pack-year. Taken together, these suggest that MARCKS is activated in response to cigarette smoke and is persistent in lung cancer. These findings implicate that MARCKS may play a role in development and progression of smoke-associated lung cancer through its activation by cigarette smoke.

How then is MARCKS modulating cancer development and progression? Given current observations of increased MARCKS activity in smoking associated lung cancer as well as previous reports of MARCKS in other cancers along with the association of poorer outcomes in smoking-associated lung cancers 40, 41, we queried what molecular signatures were involved with cancer cell aggressiveness. Differential gene expression analysis of low invasive and highly invasive lung cancer cells (CL1-0 and CL1-5 respectively) yielded a marked NF-κB molecular signature linked to MARCKS activity in highly invasive cells. NF-κB, typically composed of p50 and p65 subunits, can be activated by a wide range of signals such as TNF-α, LPS, IL-1, or LPA. After stimulation, IκB kinases are activated and degrade the inhibitory IκB protein, freeing up NF-κB 42, 43. Of note, NF-κB plays a critical role as a master regulator of inflammation, a process that has well-established links to cancer 44, 45. Cigarette smoke has long been recognized as a significant risk factor for lung cancer through its role in mediating mutagenic DNA alterations through oxidative damage and adduction as well as being able to influence and drive chronic inflammation, playing a role in tumor development and progression 10, 46. Of these pathways, the NF-κB pathway is a central pathway intertwining inflammation, carcinogenesis and cancer progression. NF-κB has already been demonstrated to be activated by cigarette smoke and plays multiple roles in promoting stemness and inflammation in cancers 47-50. Despite this and the wide-ranging roles it plays in cancer, the role of NF-κB in smoke-associated lung cancer is still not well studied.

In our study, we have found that MARCKS is associated with and modulates NF-κB activation. Phospho-MARCKS and phospho-p65 were co-upregulated in smoke-associated lung cancer specimens and tissues with high phospho-p65 levels often had higher levels of phospho-MARCKS. Moreover, in patients with high phospho-p65 and phospho-MARCKS levels had notably worse outcomes compared to patients that had only high expression of one of the proteins or low expression of both. High levels of these phospho-proteins also correlated closely with smoking status, with smokers demonstrated elevated levels of both proteins. Elevated expression of the NF-κB gene signature (TNFR, RELA, BCL2A1, CXCL1, CXCL3, LTA, RELB, and TNFα) also correlated with poorer outcomes in patients. These taken together suggests that MARCKS may be cooperating with or promoting NF-κB signaling, leading to more pronounced cancer aggressiveness as demonstrated by the poor outcomes in patients with upregulated NF-κB molecular signatures and increased phospho-MARCKS levels. Specifically, levels of MARCKS in conjunction with NF-κB pathway related genes were able to stratify overall survival as well as disease free survival and were correlated with a more aggressive cell phenotype. Given the clear stratification of patient prognosis, utilization of MARCKS with NF-κB pathway markers, such as phospho-p65, may serve as useful predictors of cancer cell behavior and outcomes in lung cancer.

As we observed elevated phospho-MARCKS in association with NF-κB activity, we next questioned the mechanism through which MARCKS is influencing NF-κB activity. To this end, we investigated the interactome of signaling molecules which may be cooperating with MARCKS to promote tumor aggressiveness. To our surprise, we noticed NF-κB-activating protein (NKAP) interacted with the unphosphorylated form of MARCKS. NKAP, as its name suggests, modulates NF-κB target gene transcription, and is mostly localized in the nucleus with a fraction being localized in the membrane 51, 52, implying that there may be spatial regulation of its function. NKAP's role in driving cancer cell growth is well established, but the regulators of this protein are not well understood 52, 53. Given its effector function is in the nucleus, it came as a surprise that we found it associated with unphosphorylated MARCKS, which is bound to the cell membrane. Assessment of downstream NF-κB activity through detecting phosphorylation and nuclear localization of p65 also indicated that upon overexpression of unphosphorylated MARCKS (S159/163A mutant MARCKS), NF-κB activity is reduced. Genetic knockdown of MARCKS or NKAP both demonstrated a similar phenomenon. Based on our observations, we proposed a possible molecular model: unphosphorylated MARCKS sequesters NKAP at the membrane, preventing its function in regulating NF-κB target gene transcription. While we did identify NKAP as a binding partner of MARCKS as well as showed that targeting of NKAP was able to reduce downstream NF-κB activity, the exact mechanisms behind how MARCKS regulates NKAP is an area in need of further investigation.

Identification of the binding sites on NKAP and MARCKS respectively as well as the exact mechanisms of how MARCKS phosphorylation promotes NKAP/NF-κB activity are primary areas that would require additional exploration. Given that phosphorylation seems to play a key role in MARCKS' ability to bind NKAP, it would be logical to predict that the interaction site for NKAP on MARCKS would likely be within its phosphorylation site domain. Further work in identifying the protein domains involved would help identify the exact mechanism regulating this interaction but is beyond the scope of this work.

As MARCKS bound to NKAP and was shown to interact with NF-κB activity, we next assessed if the activation of NF-κB activity is regulated through MARCKS phosphorylation. Upon exposure to CSE, we observed an increase of phospho-p65 concomitant with phospho-MARCKS. Pharmacologic targeting of MARCKS through a MARCKS-specific inhibitor, a small peptide that we previously developed (the MPS peptide) 13 confirmed that this activation was indeed through phospho-MARCKS. In all, we have demonstrated marked association between phospho-MARCKS and NF-κB activity in smoke-related lung cancer as well as identified MARCKS as a regulator of NF-κB through NKAP.

From a molecular perspective, we propose a novel signaling axis: the MARCKS/NKAP/p65 regulatory axis in smoke-associated lung cancer. Upon activation by cigarette smoke, MARCKS is phosphorylated, attenuating its association with NKAP, allowing NKAP to activate NF-κB signaling through phospho-p65. Activation of this pathway is indeed elevated in inflammatory disease as well as in cancers, with roles in modulating cytokines and cancer cell properties. We show that cigarette smoke is able to promote production of pro-inflammatory cytokines such as TNF-α and IL-8, which can play pro-tumor roles in the tumor microenvironment. Additionally, CSE can promote EMT markers (downregulation of E-cadherin, upregulation of Slug, fibronectin, and MMP-9) and stemness-associated genes (Sox2, OCT4, and CD133). Targeting MARCKS through MPS peptide attenuates these phenotypes. Abrogation of MARCKS activity is also able to attenuate aggressive cell behaviors including migration, invasion and oncosphere-forming ability activated by cigarette smoke. Additionally, this molecular pathway is closely associated with smoking status, pack year, as well as poor prognosis, suggesting that the pathway may be a specific driver pathway for smoking-associated lung cancer.

Although NF-κB activity is associated with cancer cell growth and aggressiveness and is elevated in smoke-associated lung cancer, NF-κB remains difficult to utilize as a drug target due to its broad regulatory functions in cells. In light of this difficulty, targeting MARCKS activity may be a viable approach as we have shown that MARCKS is able to regulate NF-κB activity instigated by cigarette smoke exposure. There is also the potential to utilize NF-κB inhibitors in conjunction with MARCKS targeting which decreases the effective dose of NF-κB inhibitors in a bid to reduce the toxicity of these therapeutics, but this area requires further investigation to assess whether combination is synergistic.

In summary, we demonstrate a novel mechanism of smoke-related lung cancer malignancy through the MARCKS-NKAP molecular complex in regulating NF-κB signaling. Inhibition of MARCKS phosphorylation is capable of trapping NKAP and inactivating NF-kB signaling, leading to suppression of proinflammatory cytokines as well as attenuation of cancer cell aggressiveness and stemness. Thus, the MARCKS/NF-κB axis presents itself as a promising biomarker, and targeting of this regulatory axis could be a viable and potential therapeutic strategy for smoke-mediated lung cancer progression.

## Supplementary Material

Supplementary figures and tables.

## Abbreviations

CSE: cigarette smoke extract
EMT: epithelial-mesenchymal transition
IHC: immunohistochemistry
MARCKS: myristoylated alanine-rich C kinase substrate
NKAP: NF-κB-activating protein
