# Supplementary material for: MARCKS cooperates with NKAP to activate NF-kB signaling in smoke-related lung cancer
Source: Theranostics. 2021 Feb 19;11(9):4122–36. doi: 10.7150/thno.53558 (PMC7977464; doi:10.7150/thno.53558)
Supplement: Supplementary file 1 — Supplementary figures and tables. [file thnov11p4122s1.pdf]

Online Data Supplement for

MARCKS cooperates with NKAP to activate NF-kB signaling in smoke-related lung cancer

Jun Liu, Szu-Jung Chen, Ssu-Wei Hsu, Jun Zhang, Ji-Min Li, David C. Yang, Shenwen Gu, Kent E. Pinkerton, Ching-Hsien Chen*

Supplementary Methods

Reagents and antibodies

RPMI-1640 medium, fetal bovine serum and penicillin-streptomycin were purchased from Life Technologies Inc. (Carlsbad, CA). Lipofect-AMINE™ was purchased from Invitrogen (Carlsbad, CA). VECTASTAIN® Elite ABC Kit (Rabbit IgG), VECTOR® Hematoxylin QS nuclear counterstain and DAB solution were purchased from VECTOR Laboratories Inc. (Burlingame, CA). Protein A-Sepharose beads and glutathione Sepharose were purchased from Amersham Biosciences (Piscataway, NJ). Anti-pSer158 MARCKS (clone EP2113Y) and anti-MARCKS (clone EP1446Y) were purchased from Abcam (Cambridge, MA). Anti-pSer159/163 MARCKS (clone D13D2), anti-pSer536 p65, anti-p65, anti-IκBα, anti-α-tubulin, Lamin B, anit-V5, GAPDH and anti-β-actin antibodies were purchased from Cell Signaling Technology, Inc. (Danvers, MA). MARCKS siRNAs (MARCKS siRNA Smartpool), NKAP siRNAs (NKAP siRNA Smartpool) and DharmaFECT siRNA transfection reagents were purchased from Dharmacon, Inc. (Lafayette, CO). Rottlerin, Gö 6976, ϵV1-2 and PKCß inhibitor were purchased from Calbiochem-EMD Millipore (Chicago, IL). The MPS peptide was purchased from EZBiolab Inc. (Carmel, IN) at a purity of 95%. The MPS peptide consisted of amino acids 151 to 175 from the wild-type protein, KKKKKRFSFKKSFKLSGFSFKKNKK. Peptide was reconstituted in phosphate-buffered saline, yielding stock concentrations of 10 mM. Stock solutions were stored at -20 °C and diluted to desired concentrations on the day of the experiment.

Plasmid constructs

For identification and cloning of the MARCKS full-length cDNA, total RNA was isolated from CL1-5 cells using Trizol reagent (Life Technologies). First-strand cDNA was reverse-transcribed with SuperScript II reverse transcriptase (Life Technologies) and oligo-dT primer. The MARCKS coding region was amplified by polymerase chain reaction (PCR) using the

forward primer: 5'-GATCCATGGGTGCCCAGTTCTCCAAGACCGCAGC-3', which introduced a BamHI site, and the reverse primer: 5'-TCTAGACTCTCTGCCGCCT CCGCTGGGGGGGCT-3', which introduced an XbaI site. The amplified product was cloned into pcDNA3.1 vector (Invitrogen). The cDNA was then fully sequenced to ensure that no mutations were introduced during the PCR amplification. For generation of MARCKS shRNA plasmids, the oligonucleotide of shRNAs (shRNA-a: 5'-GAGAAGGCGGTGAGGCTGA-3' and its complementary strand: 5'-TCAGCCTCACCGCCTTCTC-3'; shRNA-b: 5'-GAAGGTAAACGGCGACGCT-3' and its complementary strand: 5'-AGCGTCGCCGTTTACCTTC-3'; shRNA-c: 5'-GAGCGCTTCTCCTTCAAGAA-3' and its complementary strand: 5'-TTCTTGAAGGAGAAGCGCTC-3') were synthesized, annealed and cloned into the pGreenPuro shRNA expression lentivector (System Biosciences, Mountain View, CA). The S159/163A V5-tagged MARCKS was generated by site-directed mutagenesis and the mutagenic primers used were as follows: the S159A forward primer 5'-GAAGCGCTTTGCCTTCAAGAAGTCTTTCAAGCTGA-3' and the reverse primer 5'-TCAGCTTGAAAGACTTCTTGAAGCAAAGCGCTTC -3'; the S163A forward primer 5'-GAAGCCTTTTCCTTCAAGAAGGCTTTCAAGCTGA-3' and the reverse primer 5'-TCAGCTTGAAAGCCTTCTTGAAGGAAAAGCGCTTC-3'. The desired mutations were confirmed by sanger sequencing.

Cell culture and transfection

The human lung cancer cell lines, CL1-0 and CL1-5 were established as previously described [1]. The lung cancer cell line H292 and A549 were purchased from the American Type Culture Collection (ATCC) (Manassas, VA). The human HBE1 cell line was a gift from JR Yankaskas, University of North Carolina. Cancer cell lines were cultured in RPMI-1640 medium with 10% fetal bovine serum and 1% penicillin-streptomycin at 37 °C in a humidified atmosphere of 5% CO_2. Human primary bronchial epithelial cells were grown in Clonetics

BEGM medium (Cambrex Lonza, East Rutherford, New Jersey) with all hormones/growth factors included in the package, except the retinoic acid. For siRNAs transfection, ON-TARGET plus siRNA and scrambled siRNA sequences (Thermo Scientific, Pittsburgh, PA) were transfected using DharmaFECT according to the manufacturer's protocol. For enforced expression of V5-tagged MARCKS in CL1-0, lung cancer cells were transfected with pcDNA3.1-MARCKS, pcDNA3.1-S159/163A MARCKS or pcDNA3.1 vector using lipofectamine reagent (Invitrogen), according to the manufacturer's protocol. After 48 hours of transfection, cells were collected and subjected for further studies.

Exposure of cultured cells to cigarette smoke extract

Cultures of cells were exposed to cigarette smoke extract (CSE) using a protocol similar to that previously described [2]. Briefly, research cigarettes (Kentucky Tobacco R&D Center, Lexington, KY) were lit, and mainstream smoke was suctioned with a 60-ml catheter tip syringe containing 5 ml of medium. The medium was then shaken vigorously for 20 seconds. This procedure was repeated four times. The resulting medium was sterilized through a 0.22-μm filter and designated as 100% CSE. Dilutions were produced for the appropriate concentrations in treatments, as depicted in the figures. Control media were prepared similarly, except with filtered air instead of cigarette smoke.

Non-human primates of cigarette smoke exposure

The paraffin-embedded specimens of rhesus macaques exposed to filtered air (FA; control) and environmental tobacco smoke (ETS) were kindly provided from Dr. Kent E. Pinkerton (Center for Health and the Environment, UC Davis). Rhesus macaques were from the California National Primate Research Center (Davis, California). Postnatal smoking exposure was performed according to previous study [3]. Briefly, the macaques at the age of 6 months were started to be continuously exposed to filtered air (FA) or environmental tobacco smoke (ETS) for 6 months. Monkeys received 1 mg/m^3 smoking concentrate for 6 hours per day, 5 days per

week. After the period of smoke exposure, the macaques were sacrificed and the nicotine and cotinine levels in plasma were detected for confirmation. The lung tissues were collected and subjected to immunohistochemistry.

Patient tumor specimens and immunohistochemical staining

Lung tumor specimens were obtained from patients with histologically confirmed lung cancer who underwent surgical resection at the UC Davis Medical Center. None of the patients had received pre-operative adjuvant chemotherapy or radiation therapy. This investigation was approved by the Institutional Review Board of the UC Davis Health System. Written informed consent was obtained from all patients. The clinical and pathologic features of the patients and tissues are shown in Supplementary Tables S2-S7. Formalin-fixed and paraffin-embedded specimens were used, and immunohistochemical staining was performed for phospho-MARCKS and phospho-p65 levels as described previously [1, 4, 5]. Detailed experimental procedures were modified from the paraffin immunohistochemistry protocol supplied by the manufacturer (Cell Signaling, Danvers, MA). The slides were de-paraffinized in xylene and rehydrated in graded alcohol and water. An antigen retrieval step (10 nM sodium citrate (pH 6.0) at a sub-boiling temperature) was used for each primary antibody. Endogenous peroxidase activity was blocked by 3% hydrogen peroxide followed by blocking serum and incubation with appropriate antibodies overnight at 4 °C. Detection of immunostaining was carried out by using the VECTASTAIN® ABC system, according to the manufacturer's instructions (Vector Laboratories, Burlingame, CA). A four-point staining intensity scoring system was devised to confirm the relative expression of phospho-MARCKS and phospho-p65 in cancer specimens; scores ranged from zero (no expression) to 3 (highest-intensity staining) as we reported previously [1, 4, 5]. The results were classified into two groups according to the intensity and extent of staining: in the low-expression group, staining was observed in 0–1% of the cells (staining intensity score = 0), or in less than 10% of the cells (staining intensity score =1); in

the high-expression group, staining was present in 10%–50% of the cells (staining intensity score = 2), or more than 50% of the cells (staining intensity score = 3). These results were also reviewed and scored independently by two pathologists.

Western blot, immunoprecipitation, and immunofluorescent staining assays

The preparation of whole-cell lysates, cytoplasmic and nuclear extracts, and Western blot analysis was described previously [1, 5-7]. Cells were lysed in lysis buffer (50 mM Tris/HCl (pH 7.4), 1% Triton-X 100, 10% glycerol, 150 mM NaCl, 1 mM EDTA, 20 μg/ml leupeptin, 1 mM PMSF, 20 μg/mL aprotinin and 20 μg/mL pepstatin) and cleaned by pre-incubation with protein A-Sepharose beads to remove non-specifically bound proteins. After precipitation with appropriate antibodies and protein A-Sepharose beads, the immunoprecipitated complexes were washed and separated by SDS-PAGE. Immunoblotting was done with appropriate antibodies using the Amersham Biosciences enhanced chemiluminescence system for detection. For immunofluorescent staining, cells cultured on 12 mm glass cover-slips were fixed for 15 minutes in phosphate-buffered saline containing 4% paraformaldehyde and 2% sucrose and then permeabilized in phosphate-buffered saline containing 0.3% Triton X-100 for 2 minutes. Cover-slips were reacted with primary antibodies against MARCKS and NKAP as well as Alex Fluor 488 and Alex Fluor 555-labeled secondary antibodies. F-actin was stained with TRITC-conjugated phalloidin, and nuclei were demarcated with DAPI staining. The cells were mounted onto slides and visualized using fluorescence microscopy (model Axiovert 100; Carl Zeiss, Oberkochen, Germany) or a Zeiss LSM510 laser-scanning confocal microscope image system.

Flow cytometry analysis and *in vivo* tail vein metastasis assays

For flow cytometry analysis, A549 cells were harvested from either adherent or oncosphere culture medium after 14 days of culturing. Dissociated cells were stained with a combination of the following antibodies obtained from Biolegend (San Diego, CA) including

Brilliant Violet 605-conjugated anti-CD44, Alexa Fluor 488-conjugated anti-SOX2, Alexa Fluor 647-conjugated anti-Nanog, Brilliant Violet 421-conjugated anti-Oct4 antibody. Ghost Dye Violet 510 was used to discriminate the live and dead cells. The samples were analyzed by flow cytometry using a CytomicsTM FC500 flow cytometer (Beckman Coulter), according to the manufacturer's protocol. Data were acquired and analyzed using a LSR II and Flowjo software (BD Biosciences). For an *in vivo* tail vein metastasis assay, a single-cell suspension containing 1 x 10^6 adherent or oncosphere A549 cells in 0.1 mL of PBS was injected into the lateral tail veins of six-week-old NOD SCID mice (7 mice per group) purchased from Charles River Laboratories (San Diego, CA). After 14 days of tumour implantation, mice were intraperitoneally injected with either PBS or MPS peptide (28 mg/kg) every two days for 21 days and then sacrificed. The lungs and livers of these mice were removed and fixed in 4% paraformaldehyde for histological analysis. Animal usage protocols were periodically reviewed and approved by Institutional Animal Care and Use Committee at UC Davis.

Quantitative real-time PCR and RNA sequencing analysis

The mRNA expression level of target genes was detected by real-time reverse transcription polymerase chain reaction (RT-qPCR) using primers as described in the Supplementary Table 1. The house keeping gene TATA-box binding protein (TBP) was used as the reference gene. The relative expression level of target genes compared with that of TBP was defined as $-\Delta CT = -[CT_{target} - CT_{TBP}]$. The target/TBP mRNA ratio was calculated as $2^{-\Delta CT} \times K$, where K is a constant. For transcriptome profiling analysis, total cellular RNA was extracted from CL1-0 and CL1-5 cells using TRIzol reagent (Invitrogen), respectively. RNA concentration was determined using the NanoDrop machine (Thermo Fisher Scientific). Complementary DNA (cDNA) was generated using the SuperScript III First Strand cDNA Synthesis Kit (Thermo Fisher Scientific). Transcriptomic profiles were aligned to human genome using Affymetrix gene expression arrays. Differential gene expression analysis was performed using the R

package DESeq2. Genes with false discovery rate (FDR) lower than 0.05 and fold change higher than 5.0 were considered as the significantly differentially expressed genes (DEGs). Kyoto Encyclopedia of Genes and Genomes (KEGG) pathway analysis was performed on a list of DEGs using the DAVID Bioinformatics resources database.

Statistical analysis

Data are presented either as the mean ± SEM of at least three independent experiments. The quantitative *in vitro* and *in vivo* data were analyzed using the student's t-test. The difference in patient characteristics between the high-level and the low-level groups was analyzed using Fisher's exact test. In survival analysis, overall survival curves for groups with low versus high levels of phospho-MARCKS and phospho-p65 were obtained by the Kaplan–Meier, method and the differences in survival between high-level and the low-level patients were analyzed using the log-rank test. All analyses were performed using SPSS software (v20.0; SPSS, Inc., Chicago, IL). All statistical tests were two-sided and p values < 0.05 were considered statistically significant.

Supplementary Data

Table S1. PCR primers

Gene	Sequence
MARCKS	F: 5’-TTGTTGAAGAAGCCAGCATGGGTG-3’
	R: 5’-TTACCTTCACGTGGCCATTCTCCT-3’
IL-1β	F: 5'-ATTCATCCTGAATGACGCCT-3'
	R: 5'-ACCCATGTCAAATTTCACTGCTT-3'
TNF-α	F: 5'-CTGGAAAGGACACCATGAGCACT-3'
	R: 5'-TTGATGGCAGAGAGGAGGTTGAC-3'
IL-6	F: 5'-TGACAAACAAATTCGGTACATCCT-3'
	R: 5'-AGTGCCTCTTTGCTGCTTTCAC-3'
IL-8	F: 5'-ACATGACTTCCAAGCTGGCCGTGG-3'
	R: 5'-GTATGTTCTGGATATTTCATGGTAC-3'
MMP9	F: 5'-GGGACGCAGACATCGTCATC-3'
	R: 5'-TCGTCATCGTCGAAATGGGC-3'
E-cadherin	F: 5'-TCCATTTCTTGGTCTACGCC-3'
	R: 5'-CACCTTCAGCCAACCTGTT-3'
Slug	F: 5’-TGTTGC AGTGAGGGCAAGAA-3’
	R: 5’- GACCCTGGTTGCTTCAAGGA-3’
Fibronectin	F: 5'-TGGTGGCCACTAAATACGAA-3’
	R: 5'-GGAGGGCTAACATTCTCCAG-3’
OCT3/4	F: 5’-TGGGCTCGAGAAGGATGTG-3’
	R: 5’-GCATAGTCGCTGCTTGATCG-3’
SOX2	F: 5’-CACATGAAGGAGCAC CCGGATTAT-3’
	R: 5’-GTTCATGTGCGCGTAACTGTCCAT-3’
Nanog	F: 5’-AAACTATCCATCCTTGCAAATG-3’
	R: 5’-AGGAGGGAAGAGGAGACAGT-3’
CD133	F: 5’-TCCACAGAAATTT ACCTACATTGG-3’
	R: 5’-CAGCAGAGAGCAGATGACCA-3’
TBP	F: 5'-CACGAACCACGGCACTGATT-3’
	R: 5'-TTTTCTTGCTGCCAGTCTGGAC-3’
TNF-alpha	F: 5'-CTCTTCTGCCTGCTGCACTTTG-3’
	R: 5'-ATGGGCTACAGGCTTGTCACTC-3’
CXCL1	F: 5'- CTTGCCTCAATCCTGCATCC-3’
	R: 5'- CTCTGCAGCTGTGTCTCTCT-3’
CXCL3	F: 5'- CAAACCGAAGTCATAGCCACAC-3’
	R: 5'-ACCCTGCAGGAAGTGTCAATG-3’

BCL2A	F: 5'-GCCCACAAGAAGAGGAAAATGG-3’
	R: 5'-TGGAGTGTCCTTTCTGGTCA-3’
TNFR1A	F: 5'-GACTGCAGGGAGTGTGAGAG-3’
	R: 5'-CCTGACCCATTTCCTTTCGG-3’

Table S2. phospho-MARCKS levels in relation to clinicopathologic characteristics of 141 lung cancer patients

Characteristic	Total patients	High No. of Patients (%)	Low No. of Patients (%)	*p*-value
**Number of patients**	n=141	n=53	n=88	
**Age (mean ± SD)**	66.64±10.08	67.10±9.81	66.36±10.29	0.674†
**Gender**				0.729‡
Male	61	24 (45.3)	37 (42.0)	
Female	80	29 (54.7)	51 (58.0)	
**Race**				0.070‡
White	86	35 (66.1)	51 (57.9)	
Black	7	4 (7.5)	3 (3.4)	
Asian	6	4 (7.5)	2 (2.3)	
Other	42	10 (18.9)	32 (36.4)	
**Grade***				0.169‡
1: Well	22	8 (17.8)	14 (21.2)	
2: Moderate	39	12 (26.7)	27 (40.9)	
3: Poor	50	25 (55.5)	25 (37.9)	
**Type**				0.119‡
Adenocarcinoma	65	26 (49.0)	39 (48.75)	
Squamous cell carcinoma	47	18 (34.0)	29 (36.25)	
Large cell carcinoma	10	6 (11.3)	4 (5.0)	
Bronchioloalveolar carcinoma	7	0 (0.0)	7 (8.75)	
Other	12	3 (5.7)	9 (11.25)	

† Student T-test,

‡ Fisher exact test.

*Some patients without grade information.

Table S3. phospho-p65 levels in relation to clinicopathologic characteristics of 141 lung cancer patients

Characteristic	Total patients	High No. of Patients (%)	Low No. of Patients (%)	*p*-value
**Number of patients**	n=141	n=71	n=70	
**Age (mean ± SD)**	66.64±10.08	67.12±9.99	66.15±10.23	0.571†
**Gender**				0.062‡
Male	61	25 (35.2)	36 (51.4)	
Female	80	46 (64.8)	34 (48.6)	
**Race**				0.500‡
White	86	46 (64.8)	40 (57.2)	
Black	7	2 (2.8)	5 (7.1)	
Asian	6	2 (2.8)	4 (5.7)	
Other	42	21 (29.6)	21 (30.0)	
**Grade***				0.939‡
1: Well	22	10 (18.5)	12 (21.0)	
2: Moderate	39	19 (35.2)	20 (35.1)	
3: Poor	50	25 (46.3)	25 (43.9)	
**Type**				0.345‡
Adenocarcinoma	65	28 (39.4)	37 (52.9)	
Squamous cell carcinoma	47	24 (33.8)	23 (32.9)	
Large cell carcinoma	10	7 (9.9)	3 (4.2)	
Bronchioloalveolar carcinoma	7	5 (7.0)	2 (2.9)	
Other	12	7 (9.9)	5 (7.1)	

† Student T-test,

‡ Fisher exact test.

* Some patients without grade information.

Table S4. Clinicopathologic characteristics of 141 lung cancer patients#

Case ID	Age (years)	Gender	Race	Grade	Type	OS/ Mon	OS/ Censor	DFS/ Mon	DFS/ Censor	p-MARCKS (H: 1)	p-p65 (H: 1)
1	80	M	Caucasian		Squamous Cell Carcinoma	22	1	22	1	0	1
2	77	F	Caucasian		Adenocarcinoma	120	0	120	0	0	0
3	72	F	Caucasian	Well	Squamous Cell Carcinoma	120	0	120	0	1	0
4	60	F	Black	Poor	Adenocarcinoma	120	0	120	0	1	0
5	61.5	F	Caucasian	Moderate	Adenocarcinoma	14	1	14	1	0	1
6	70	F	Other	Moderate	Squamous Cell Carcinoma	30	1	30	1	0	1
7	78	M	Caucasian	Well	Squamous Cell Carcinoma	95	1	94	1	1	1
8	82	F	Other	Moderate	Squamous Cell Carcinoma	120	0	120	0	0	0
9	61.5	M	Other	Well	Adenocarcinoma	75	1	75	1	0	0
10	64	F	Other	Moderate	Adenocarcinoma	120	0	120	0	0	1
11	52	F	Other	Moderate	Squamous Cell Carcinoma	120	0	120	0	0	1
12	52	M	Other	Poor	Squamous Cell Carcinoma	120	0	120	0	0	0
13	56	F	Other	Poor	Adenocarcinoma	73	1	73	1	1	1
14	77	F	Caucasian	Moderate	Adenocarcinoma	9	1	9	1	1	0
15	53	M	Caucasian	Moderate	Adenocarcinoma	42	1	39	1	0	0
16	71	M	Caucasian	Moderate	Squamous Cell Carcinoma	120	0	120	0	1	0
17	59	F	Caucasian		Adenocarcinoma	104	1	104	1	0	1
18	72	F	Other	Poor	Squamous Cell Carcinoma	120	0	109	1	0	1
19	32.5	F	Caucasian	Poor	Other					0	1
20	57	F	Other		Large Cell Carcinoma	120	0	120	0	0	0
21	68	F	Caucasian	Well	Adenocarcinoma	120	0	120	0	0	0

22	63.5	F	Caucasian	Moderate	Squamous Cell Carcinoma	33	1	17	1	1	1
23	66.5	F	Other	Moderate	Adenocarcinoma	120	0	120	0	0	0
24	83	M	Caucasian	Poor	Squamous Cell Carcinoma	85	1	85	1	1	0
25	79.5	F	Caucasian		Other	3	1	3	1	0	1
26	65	M	Caucasian	Moderate	Adenocarcinoma	69	1	69	1	1	1
27	78	M	Caucasian	Poor	Adenocarcinoma	120	0	120	0	1	1
28	57.5	M	Caucasian	Moderate	Adenocarcinoma					0	0
29	68.5	F	Other		Brochioloalveolar Carcinoma					0	1
30	66	M	Caucasian	Moderate	Squamous Cell Carcinoma	6	1	6	1	0	1
31	79	F	Black	Moderate	Squamous Cell Carcinoma	63	1	63	1	0	0
32	59.5	M	Caucasian	Poor	Other	70	1	70	1	0	0
33	57	M	Black	Moderate	Adenocarcinoma	120	0	120	0	0	0
34	55.5	M	Caucasian	Poor	Squamous Cell Carcinoma	67	1	67	1	1	1
35	60	F	Other	Poor	Squamous Cell Carcinoma	53	1	53	1	1	1
36	47.5	M	Other	Well	Adenocarcinoma	120	0	120	0	0	0
37	65.5	F	Caucasian		Adenocarcinoma	92	1	92	1	0	1
38	71	M	Caucasian	Poor	Other	2	1	2	1	1	1
39	53.5	F	Caucasian		Adenocarcinoma	120	0	120	0	0	0
40	52	F	Caucasian		Adenocarcinoma	120	0	49	1	0	0
41	56.5	F	Other	Poor	Brochioloalveolar Carcinoma	120	0	120	0	0	1
42	80	F	Caucasian	Moderate	Squamous Cell Carcinoma	3	1	3	1	0	1
43	69	M	Caucasian	Poor	Squamous Cell Carcinoma					0	0
44	81	F	Other		Large Cell Carcinoma	120	0	120	0	0	1
45	65	F	Caucasian	Well	Adenocarcinoma	64	1	64	1	0	1

46	61	F	Caucasian	Poor	Squamous Cell Carcinoma	100	1	100	1	0	1
47	54.5	F	Caucasian	Well	Adenocarcinoma	85	1	85	1	1	1
48	74.6	F	Caucasian		Adenocarcinoma	76	1	64	1	1	1
49	76.6	M	Caucasian	Poor	Adenocarcinoma	33	1	33	1	0	1
50	59.5	F	Caucasian	Moderate	Adenocarcinoma					0	1
51	62	M	Caucasian	Well	Brochioloalveolar Carcinoma	33	1	33	1	0	0
52	65	F	Caucasian	Moderate	Squamous Cell Carcinoma	120	0	120	0	0	1
53	73.5	F	Caucasian	Poor	Squamous Cell Carcinoma	18	1	18	1	1	1
54	75	M	Other	Moderate	Adenocarcinoma					1	0
55	81	F	Caucasian	Well	Adenocarcinoma	100	1	100	1	1	1
56	75	F	Caucasian		Brochioloalveolar Carcinoma	1	1	1	1	0	1
57	58.6	F	Caucasian	Poor	Adenocarcinoma					1	1
58	59.5	F	Caucasian	Poor	Squamous Cell Carcinoma	120	0	120	0	1	0
59	70	F	Caucasian	Well	Adenocarcinoma	55	1	55	1	1	1
60	71	M	Black	Moderate	Adenocarcinoma	59	1	59	1	1	1
61	53.5	F	Caucasian	Well	Adenocarcinoma	55	1	55	1	1	0
62	59.5	M	Caucasian	Poor	Squamous Cell Carcinoma	7	1	7	1	1	1
63	68.5	M	Caucasian	Poor	Squamous Cell Carcinoma					0	0
64	67.2	M	Other	Moderate	Squamous Cell Carcinoma	120	0	120	0	0	1
65	81	M	Other		Other	25	1	25	1	0	1
66	55	M	Caucasian	Moderate	Squamous Cell Carcinoma	120	0	120	0	0	0
67	74	M	Other	Poor	Squamous Cell Carcinoma	105	1	105	1	0	1
68	80	F	Caucasian		Adenocarcinoma	29	1	29	1	1	1
69	72	F	Caucasian	Poor	Adenocarcinoma	58	1	58	1	1	0

70	63	M	Caucasian	Well	Large Cell Carcinoma	56	1	56	1	0	1
71	60	F	Black	Poor	Adenocarcinoma					1	1
72	63.5	F	Other	Poor	Adenocarcinoma	37	1	37	1	0	1
73	60	M	Caucasian	Poor	Squamous Cell Carcinoma					1	0
74	85	M	Other	Moderate	Adenocarcinoma					0	0
75	63.5	F	Other	Moderate	Squamous Cell Carcinoma					0	0
76	73.5	F	Other	Moderate	Squamous Cell Carcinoma					1	1
77	70	M	Caucasian	Moderate	Adenocarcinoma	20	1	20	1	0	0
78	68	F	Other	Poor	Squamous Cell Carcinoma					0	1
79	76	F	Caucasian	Well	Adenocarcinoma					0	1
80	71	M	Other	Moderate	Adenocarcinoma	14	1	14	1	0	0
81	83.5	M	Caucasian	Poor	Squamous Cell Carcinoma	4	1	4	1	0	1
82	78	F	Caucasian	Poor	Squamous Cell Carcinoma	3	1	3	1	1	1
83	59	F	Caucasian		Adenocarcinoma	120	0	120	0	1	0
84	66.5	F	Caucasian		Other	2	1	2	1	0	1
85	68.2	F	Other		Adenocarcinoma	120	0	120	0	0	1
86	68	F	Other		Adenocarcinoma	24	1	24	1	0	0
87	68	M	Other	Moderate	Squamous Cell Carcinoma					0	0
88	86.6	M	Other	Moderate	Adenocarcinoma	30	1	30	1	1	1
89	69	F	Caucasian	Well	Adenocarcinoma	120	0	120	0	0	0
90	56	F	Other	Well	Adenocarcinoma	120	0	120	0	0	0
91	79	M	Caucasian	Well	Adenocarcinoma	120	0	120	0	0	0
92	57	F	Caucasian	Moderate	Squamous Cell Carcinoma	20	1	20	1	1	1
93	59	M	Black	Poor	Adenocarcinoma	23	1	23	1	0	0

94	57	F	Other	Poor	Squamous Cell Carcinoma	120	0	120	0	0	0
95	75	F	Other	Poor	Adenocarcinoma	120	0	120	0	0	0
96	75	F	Other	Poor	Adenocarcinoma	120	0	13	1	0	0
97	59	M	Black		Squamous Cell Carcinoma	120	0	120	0	1	0
98	70	F	Other	Poor	Adenocarcinoma					1	1
99	81.5	M	Other	Well	Adenocarcinoma	120	0	120	0	0	0
100	76	F	Other	Moderate	Adenocarcinoma	120	0	120	0	0	0
101	66.5	M	Other	Well	Other	120	0	22	1	0	0
102	62	F	Caucasian	Moderate	Other	120	0	25	1	1	0
103	55	M	Caucasian		Large Cell Carcinoma					1	1
104	64.5	F	Caucasian	Well	Brochioloalveolar Carcinoma	120	0	112	1	0	1
105	70	F	Caucasian	Well	Adenocarcinoma	120	0	120	0	1	1
106	71	F	Asian		Other	4	0	4	1	0	1
107	85	M	Caucasian	Poor	Large Cell Carcinoma	90	1	90	1	1	1
108	58.5	F	Caucasian	Poor	Adenocarcinoma	39	1	39	1	0	0
109	58	M	Caucasian	Poor	Large Cell Carcinoma	20	1	20	1	0	0
110	51	F	Caucasian	Moderate	Adenocarcinoma	100	1	79	1	0	0
111	72	M	Asian	Poor	Adenocarcinoma	46	1	46	1	1	0
112	51.5	M	Asian	Poor	Adenocarcinoma	15	1	15	1	1	1
113	56	M	Caucasian	Moderate	Squamous Cell Carcinoma	49	1	49	1	0	1
114	66.5	M	Other	Moderate	Other	120	0	120	0	1	1
115	60	M	Caucasian	Moderate	Squamous Cell Carcinoma	32	1	18	1	1	1
116	57	M	Other	Poor	Large Cell Carcinoma	120	0	120	0	1	1
117	58	M	Caucasian	Moderate	Squamous Cell Carcinoma	93	1	93	1	0	1

118	74	F	Caucasian	Poor	Squamous Cell Carcinoma	26	1	26	1	0	0
119	82	M	Caucasian	Poor	Squamous Cell Carcinoma	77	1	77	1	1	0
120	65	M	Caucasian	Poor	Adenocarcinoma					0	0
121	73.5	M	Caucasian	Poor	Adenocarcinoma					0	0
122	45	M	Asian	Poor	Adenocarcinoma	25	1	25	1	1	0
123	85	M	Caucasian	Well	Squamous Cell Carcinoma					0	0
124	41	F	Caucasian		Other	120	0	120	0	0	0
125	68	F	Caucasian	Well	Adenocarcinoma	31	1	31	1	1	1
126	68	M	Asian		Other	120	0	120	0	0	0
127	69.5	M	Asian		Large Cell Carcinoma	11	1	11	1	1	0
128	77	F	Other		Squamous Cell Carcinoma	27	1	27	1	0	0
129	74	M	Other	Poor	Squamous Cell Carcinoma	120	0	120	0	1	0
130	60.5	F	Caucasian	Poor	Adenocarcinoma	18	1	18	1	0	0
131	65.5	F	Caucasian	Poor	Adenocarcinoma					0	0
132	71	F	Caucasian		Large Cell Carcinoma	77	1	77	1	1	1
133	74	F	Other	Poor	Large Cell Carcinoma	40	1	40	1	1	1
134	51	F	Caucasian	Poor	Adenocarcinoma	24	1	24	1	1	1
135	71	F	Caucasian	Poor	Squamous Cell Carcinoma	77	1	77	1	0	0
136	77	M	Caucasian	Moderate	Squamous Cell Carcinoma	120	0	120	0	0	0
137	80	F	Caucasian	Moderate	Squamous Cell Carcinoma	120	0	120	0	0	0
138	56	M	Caucasian		Brochioloalveolar Carcinoma	36	1	36	1	0	0
139	54	M	Caucasian		Adenocarcinoma					0	1
140	67	F	Caucasian		Adenocarcinoma	37	1	31	1	1	0
141	77	F	Caucasian		Brochioloalveolar Carcinoma	120	0	120	0	0	1

[#] 23 patients without survival information.

Table S5. phospho-MARCKS levels in relation to clinicopathologic characteristics of 96 lung cancer patients

Characteristic	Total patients	High No. of Patients (%)	Low No. of Patients (%)	*p*-value
**Number of patients**	n=96	n=54	n=42	
**Age (mean ± SD)**	67.08±9.67	68.24±9.67	65.60±9.58	0.186†
**Gender**				0.413‡
Male	42	21 (38.9)	20 (47.6)	
Female	54	33 (61.1)	22 (52.4)	
**Race**				0.468‡
White	54	27 (50.0)	27 (64.2)	
Black	5	4 (7.4)	1 (2.4)	
Asian	5	3 (5.6)	2 (4.8)	
Other	32	20 (37.0)	12 (28.6)	
**Smoke history#**				0.003‡
Yes	68	45 (83.3)	23 (54.8)	
No	28	9 (16.7)	19 (45.2)	
**Grade***				0.094‡
1: Well	10	4 (9.1)	6 (18.7)	
2: Moderate	29	14 (31.8)	15 (46.9)	
3: Poor	37	26 (59.1)	11 (34.4)	
**Type**				0.774‡
Adenocarcinoma	46	25 (46.3)	21 (50.0)	
Squamous cell carcinoma	31	18 (33.3)	13 (31.0)	
Large cell carcinoma	5	4 (7.4)	1 (2.4)	
Bronchioloalveolar carcinoma	5	2 (3.7)	3 (7.1)	
Other	9	5 (9.3)	4 (9.5)	

† Student T-test,

‡ Fisher exact test.

Some patients without pack year information

* Some patients without grade information

Table S6. phospho-p65 levels in relation to clinicopathologic characteristics of 96 lung cancer patients

Characteristic	Total patients	High<br>No. of Patients (%)	Low<br>No. of Patients (%)	*p*-value
**Number of patients**	n=96	n=46	n=50	
**Age (mean ± SD)**	67.08±9.67	68.25±8.53	66.01±10.58	0.260†
**Gender**				0.307‡
Male	42	17 (37.0)	24 (48.0)	
Female	54	29 (63.0)	26 (52.0)	
**Race**				0.884‡
White	54	25 (54.4)	29 (58.0)	
Black	5	2 (4.3)	3 (6.0)	
Asian	5	2 (4.3)	3 (6.0)	
Other	32	17 (37.0)	15 (30.0)	
**Smoke history#**				0.001‡
Yes	68	40 (87.0)	28 (56.0)	
No	28	6 (13.0)	22 (44.0)	
**Grade***				0.592‡
1: Well	10	3 (8.8)	7 (16.7)	
2: Moderate	29	14 (41.2)	15 (35.7)	
3: Poor	37	17 (50.0)	20 (47.6)	
**Type**				0.251‡
Adenocarcinoma	46	17 (37.0)	29 (58.0)	
Squamous cell carcinoma	31	17 (37.0)	14 (24.0)	
Large cell carcinoma	5	4 (8.7)	1 (2.0)	
Bronchioloalveolar carcinoma	5	3 (6.5)	2 (4.0)	
Other	9	5 (10.8)	4 (8.0)	

† Student T-test.

‡ Fisher exact test.

Some patients without pack year information

* Some patients without grade information.

Table S7. Clinicopathologic characteristics of 96 lung cancer patients#

Case ID	Smoking	Age (years)	Gender	Race	Grade	Type	OS/ Mon	OS/ Censor	DFS/ Mon	DFS/ Censor	p-MARCKS (H:1)	p-p65 (H:1)
1	No	77	F	Caucasian		Adenocarcinoma	120	0	120	0	0	0
2	Yes	70	F	Other	Moderate	Squamous Cell Carcinoma	30	1	30	1	0	1
3	Yes	78	M	Caucasian	Well	Squamous Cell Carcinoma	95	1	94	1	1	1
4	No	82	F	Other	Moderate	Squamous Cell Carcinoma	120	0	120	0	0	0
5	No	64	F	Other	Moderate	Adenocarcinoma	120	0	120	0	1	1
6	No	52	F	Other	Moderate	Squamous Cell Carcinoma	120	0	120	0	0	1
7	Yes	52	M	Other	Poor	Squamous Cell Carcinoma	120	0	120	0	0	0
8	Yes	53	M	Caucasian	Moderate	Adenocarcinoma	42	1	39	1	0	0
9	Yes	72	F	Other	Poor	Squamous Cell Carcinoma	120	0	109	1	1	1
10	Yes	57	F	Other		Large Cell Carcinoma					1	0
11	No	68	F	Caucasian	Well	Adenocarcinoma	120	0	120	0	0	0
12	Yes	66.5	F	Other	Moderate	Adenocarcinoma					0	0
13	No	83	M	Caucasian	Poor	Other	85	1	85	1	1	0
14	Yes	79.5	F	Caucasian		Other	3	1	3	1	1	1
15	Yes	65	M	Caucasian	Moderate	Adenocarcinoma	69	1	69	1	1	1
16	Yes	57.5	M	Caucasian	Moderate	Adenocarcinoma					0	0
17	Yes	66	M	Caucasian	Moderate	Squamous Cell Carcinoma	6	1	6	1	1	1
18	Yes	79	F	Black	Moderate	Squamous Cell Carcinoma	63	1	63	1	1	0
19	No	59.5	M	Caucasian	Poor	Other	70	1	70	1	0	0
20	Yes	57	M	Black	Moderate	Adenocarcinoma					0	0

21	Yes	60	F	Other	Poor	Other	53	1	53	1	1	1
22	No	65.5	F	Caucasian		Adenocarcinoma	92	1	92	1	0	1
23	No	53.5	F	Caucasian		Adenocarcinoma	120	0	120	0	0	0
24	Yes	52	F	Caucasian		Adenocarcinoma					1	0
25	No	69	M	Caucasian	Poor	Squamous Cell Carcinoma					0	0
26	Yes	81	F	Other		Large Cell Carcinoma	120	0	120	0	1	1
27	Yes	61	F	Caucasian	Poor	Squamous Cell Carcinoma	100	1	100	1	0	1
28	Yes	74.6	F	Caucasian		Adenocarcinoma	76	1	64	1	1	1
29	No	76.6	M	Caucasian	Poor	Adenocarcinoma	33	1	33	1	0	1
30	Yes	59.5	F	Caucasian	Moderate	Adenocarcinoma	120	0	120	0	0	1
31	No	62	M	Caucasian	Well	Brochioloalveolar Carcinoma	33	1	33	1	0	0
32	Yes	73.5	F	Caucasian	Poor	Squamous Cell Carcinoma	18	1	18	1	1	1
33	Yes	75	F	Caucasian		Brochioloalveolar Carcinoma	1	1	1	1	1	1
34	Yes	58.6	F	Caucasian	Poor	Adenocarcinoma	120	0	120	0	1	1
35	Yes	59.5	F	Caucasian	Poor	Squamous Cell Carcinoma					1	0
36	Yes	71	M	Black	Moderate	Adenocarcinoma	59	1	59	1	1	1
37	Yes	59.5	M	Caucasian	Poor	Squamous Cell Carcinoma	7	1	7	1	1	1
38	Yes	68.5	M	Caucasian	Poor	Squamous Cell Carcinoma	0.3	1	0.3	1	1	0
39	Yes	67.2	M	Other	Moderate	Squamous Cell Carcinoma	120	0	120	0	1	1
40	Yes	81	M	Other		Other	25	1	25	1	0	1
41	Yes	55	M	Caucasian	Moderate	Squamous Cell Carcinoma					0	0
42	Yes	74	M	Other	Poor	Squamous Cell Carcinoma	105	1	105	1	1	1
43	No	72	F	Caucasian	Poor	Adenocarcinoma	58	1	58	1	1	0
44	No	63	M	Caucasian	Well	Large Cell Carcinoma	56	1	56	1	0	1

45	Yes	60	F	Black	Poor	Adenocarcinoma					1	1
46	Yes	63.5	F	Other	Poor	Adenocarcinoma	37	1	37	1	1	1
47	Yes	85	M	Other	Moderate	Adenocarcinoma	3	1	3	1	0	0
48	Yes	63.5	F	Other	Moderate	Squamous Cell Carcinoma	5	1	5	1	1	0
49	Yes	73.5	F	Other	Moderate	Squamous Cell Carcinoma	120	0	120	0	1	1
50	Yes	70	M	Caucasian	Moderate	Adenocarcinoma	20	1	20	1	1	0
51	Yes	68	F	Other	Poor	Squamous Cell Carcinoma	120	0	120	0	0	1
52	Yes	83.5	M	Caucasian	Poor	Squamous Cell Carcinoma	4	1	4	1	1	1
53	Yes	78	F	Caucasian	Poor	Squamous Cell Carcinoma	3	1	3	1	1	1
54	No	59	F	Caucasian		Adenocarcinoma	120	0	120	0	1	0
55	Yes	66.5	F	Caucasian		Other	2	1	2	1	0	1
56	Yes	68.2	F	Other		Adenocarcinoma	120	0	120	0	1	1
57	No	68	F	Other		Adenocarcinoma	24	1	24	1	0	0
58	Yes	68	M	Other	Moderate	Squamous Cell Carcinoma	9	1	9	1	1	0
59	Yes	86.6	M	Other	Moderate	Adenocarcinoma	30	1	30	1	1	1
60	Yes	69	F	Caucasian	Well	Adenocarcinoma					0	0
61	Yes	56	F	Other	Well	Adenocarcinoma					0	0
62	Yes	79	M	Caucasian	Well	Adenocarcinoma					0	0
63	Yes	57	F	Caucasian	Moderate	Squamous Cell Carcinoma	120	0	120	0	1	1
64	Yes	59	M	Black	Poor	Adenocarcinoma	23	1	23	1	1	0
65	No	57	F	Other	Poor	Squamous Cell Carcinoma	120	0	120	0	0	0
66	Yes	75	F	Other	Poor	Adenocarcinoma	120	0	120	0	1	0
67	Yes	75	F	Other	Poor	Adenocarcinoma					0	0
68	Yes	70	F	Other	Poor	Adenocarcinoma					1	1

69	Yes	81.5	M	Other	Well	Adenocarcinoma					1	0
70	Yes	76	F	Other	Moderate	Adenocarcinoma					1	0
71	Yes	66.5	M	Other	Well	Brochioloalveolar Carcinoma					1	0
72	Yes	70	F	Caucasian	Poor	Adenocarcinoma					1	1
73	Yes	71	F	Asian		Brochioloalveolar Carcinoma	4	0	4	1	0	1
74	No	58.5	F	Caucasian	Poor	Adenocarcinoma	39	1	39	1	1	0
75	Yes	51	F	Caucasian	Moderate	Adenocarcinoma	100	1	79	1	0	0
76	No	72	M	Asian	Poor	Other	46	1	46	1	1	0
77	Yes	51.5	M	Asian	Poor	Adenocarcinoma	15	1	15	1	1	1
78	Yes	56	M	Caucasian	Moderate	Squamous Cell Carcinoma	49	1	49	1	0	1
79	Yes	66.5	M	Other	Moderate	Other	120	0	120	0	1	1
80	No	58	M	Caucasian	Moderate	Squamous Cell Carcinoma	93	1	93	1	0	1
81	No	82	M	Caucasian	Poor	Squamous Cell Carcinoma	77	1	77	1	1	0
82	No	65	M	Caucasian	Poor	Adenocarcinoma					0	0
83	No	73.5	M	Caucasian	Poor	Adenocarcinoma					0	0
84	No	45	M	Asian	Poor	Adenocarcinoma	25	1	25	1	1	0
85	No	41	F	Caucasian		Adenocarcinoma	120	0	120	0	1	0
86	Yes	68	F	Caucasian	Well	Adenocarcinoma	31	1	31	1	1	1
87	No	68	M	Asian		Other	120	0	120	0	0	0
88	Yes	60.5	F	Caucasian	Poor	Adenocarcinoma	18	1	18	1	0	0
89	Yes	65.5	F	Caucasian	Poor	Adenocarcinoma	8	1	8	1	1	0
90	Yes	71	F	Caucasian		Large Cell Carcinoma	77	1	77	1	1	1
91	Yes	74	F	Other	Poor	Large Cell Carcinoma	40	1	40	1	1	1
92	Yes	71	F	Caucasian	Poor	Squamous Cell Carcinoma	77	1	77	1	1	0

93	No	77	M	Caucasian	Moderate	Squamous Cell Carcinoma	120	0	120	0	0	0
94	No	80	F	Caucasian	Moderate	Squamous Cell Carcinoma	120	0	120	0	0	0
95	Yes	54	M	Caucasian		Adenocarcinoma	120	0	120	0	0	1
96	Yes	77	F	Caucasian		Brochioloalveolar Carcinoma	120	0	120	0	0	1

[#] 20 patients without survival information.

Supplementary Figure S1.

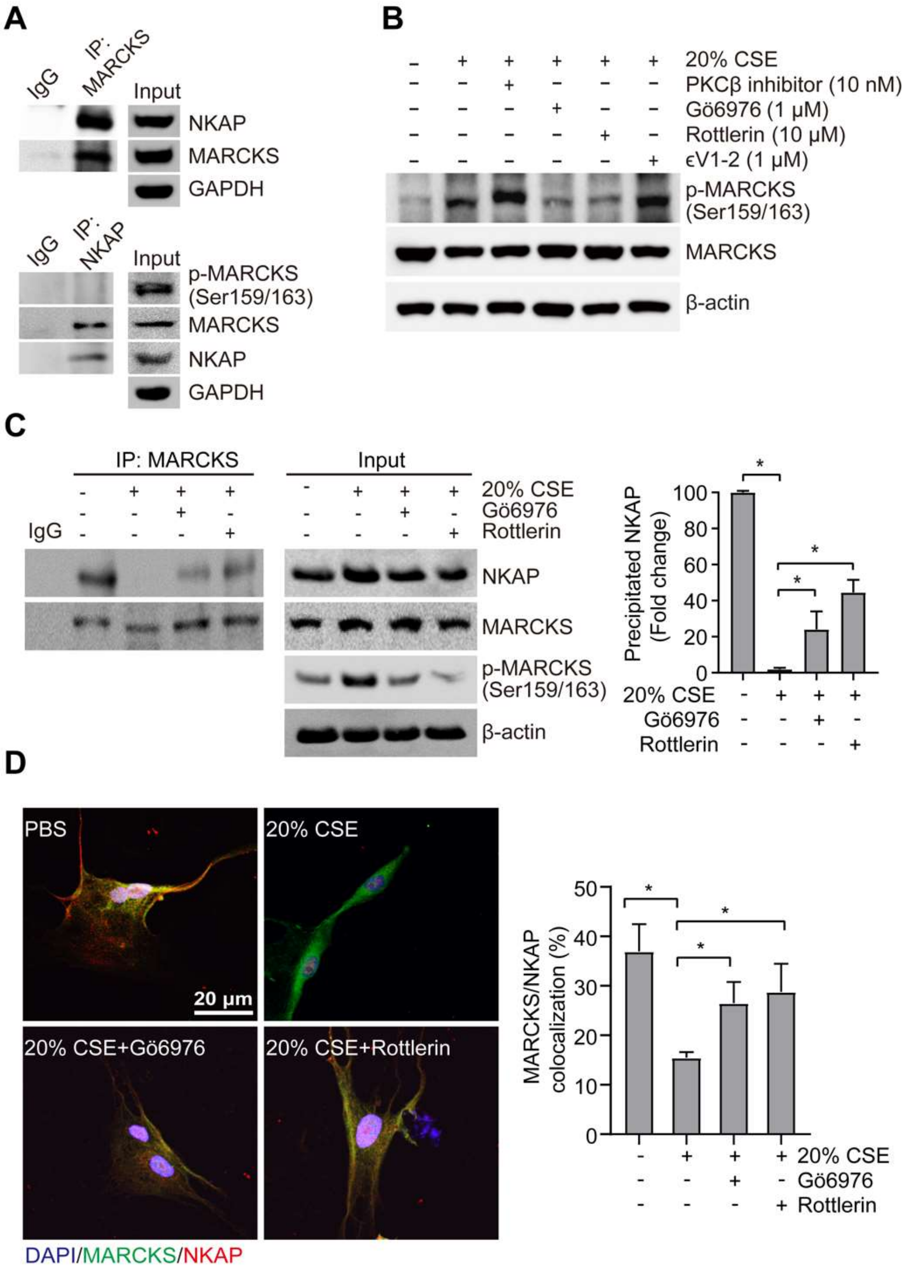

Supplementary Figure S1.

(**A**) Determination of an endogenous interaction between MARCKS and NKAP in CL1-5 cells by immunoprecipitation of MARCKS protein (Top) and NKAP (bottom). (**B**) The effects of specific PKC isoforms inhibitors on phospho-MARCKS abundance in smoke-treated cells. Cells were co-treated with 20% cigarette smoke extract (CSE) and various PKC isoforms inhibitors as indicated. The levels of phospho-MARCKS and MARCKS in the cells were determined by Western blots. Rottlerin: PKC-delta inhibitor; Gö6976: PKC-alpha inhibitor; ϵV1-2: PKC-epsilon inhibitor; PKCß: PKC-beta inhibitor. (**C**) The effects of PKC-delta inhibitor Rottlerin or PKC-alpha inhibitor Gö6976 on the interaction between MARCKS and NKAP in PBS or smoke-exposed HBE1 cells. Cells were co-treated with 20% cigarette smoke extract (CSE) and Gö6976 or Rottlerin as indicated. The amount of NKAP protein co-precipitated with MARCKS protein was analysed by using immunoblot assays. The mean results for densitometric scans of three blots from three separate experiments are shown in right panel. Data are represented as mean ± SEM; *, $p < 0.05$. (**D**) These cells as described in C were stained for MARCKS and NKAP. The fluorescence of FITC-conjugated MARCKS (green), TRITC-conjugated NKAP (red) and DAPI (nucleus counter-stained: blue) was visualized under a confocal laser-scanning microscope. Scale bar: 20 μm.

Supplementary Figure S2.

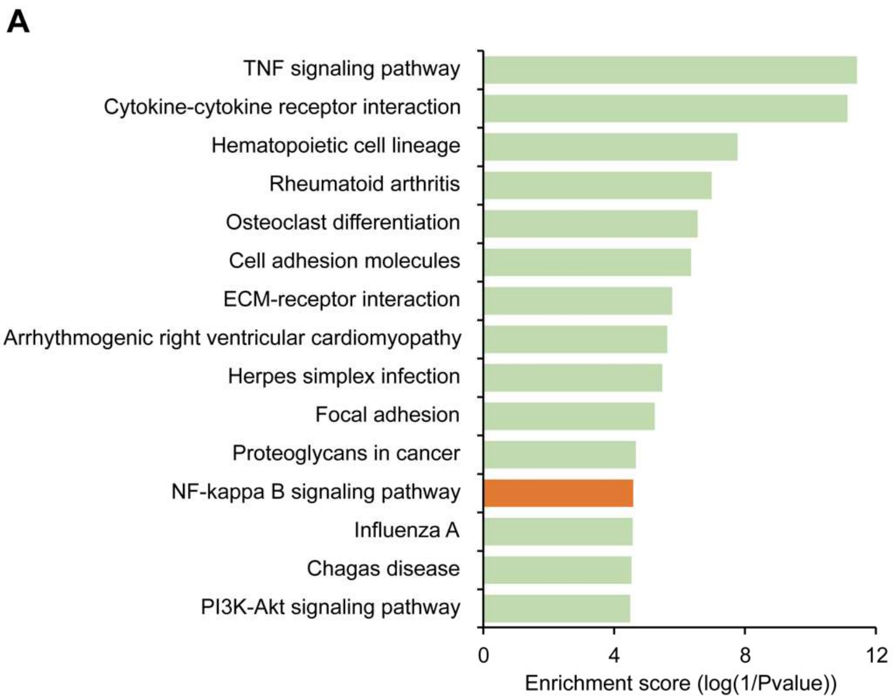

Supplementary Figure S2.

(**A**) DAVID pathway enrichment analysis of RNA-seq profiles revealed significantly enriched signaling pathways in highly phospho-MARCKS-expressing CL1-5 cells versus C1-0 cells with low phospho-MARCKS expression. The horizontal axis describes the log(1/Pvalue) of the significant pathways. The vertical axis represents the protein clusters involved in the DAVID pathways.

Supplementary Figure S3.

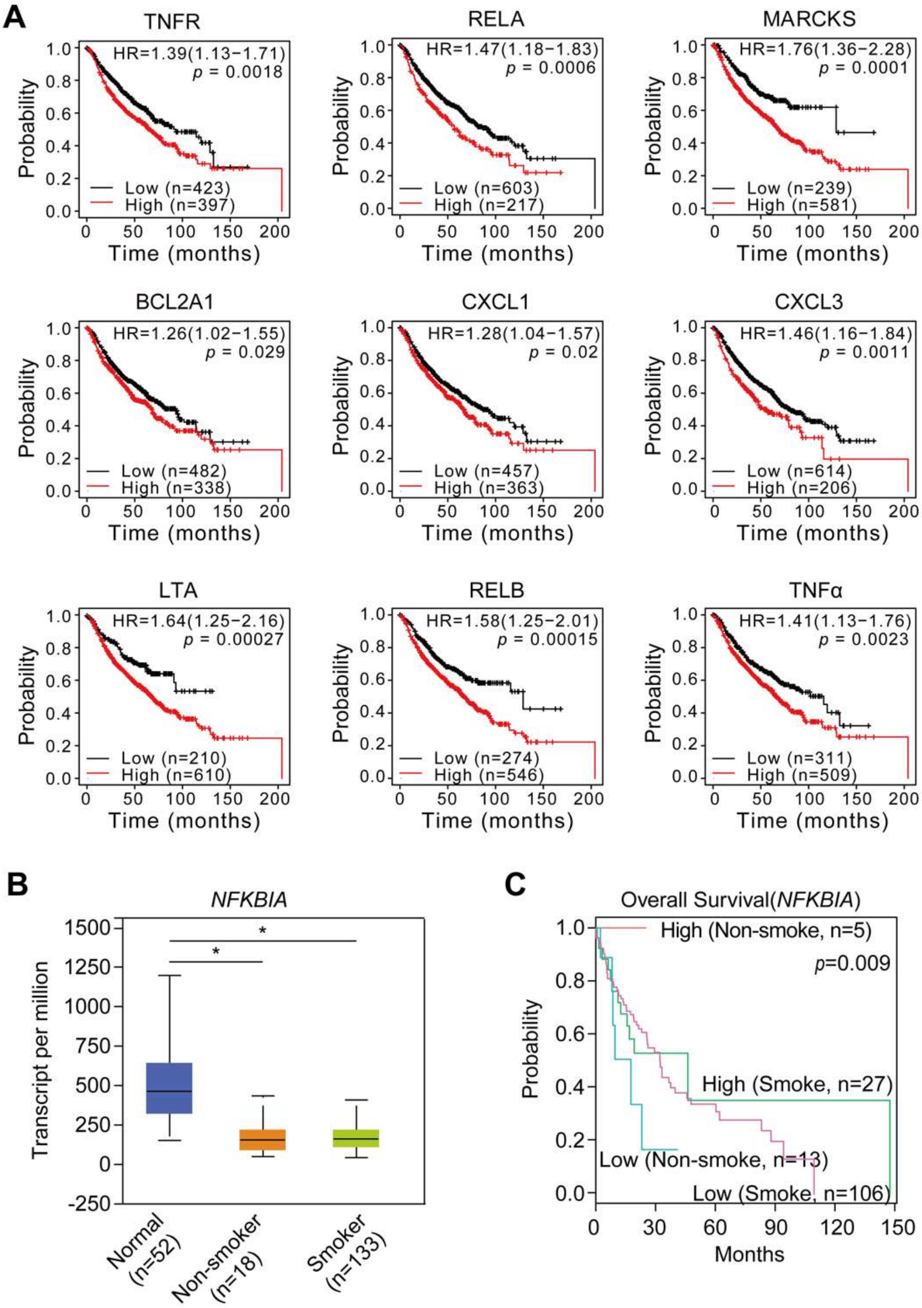

Supplementary Figure S3.

(**A**) Kaplan-Meier plot shows overall survival of a cohort of 810 smoke-related lung cancer patients from the TCGA database (https://kmplot.com/analysis/) with high or low expression of NF-κB signaling-related genes including *TNFR, RELA, MARCKS, BCL2A1, CXCL1, CXCL3, LTA, RELB* and *TNF-A* that was found to be upregulated in CL1-5 cells as compared to CL1-0 cells. The best cutoff value was automatically computed. Two-side log-rank *p* value and HR are displayed. (**B**) The transcriptional level of *NFKBIA* in smokers (n=133), non-smokers (n=18), and healthy controls (n=52) from a cohort of lung cancer patients (n=151) and healthy controls (n=52) using the TCGA database (http://ualcan.path.uab.edu/index.html). *, $p < 0.05$. (**C**) The overall survival of smokers with high (n=27) or low (n=106) expression of *NFKBIA* and non-smoker with high (*n*=5) or low (*n*=13) expression of *NFKBIA* in lung cancer patients was analyzed by the Kaplan–Meier and log-rank test.

Supplementary Figure S4.

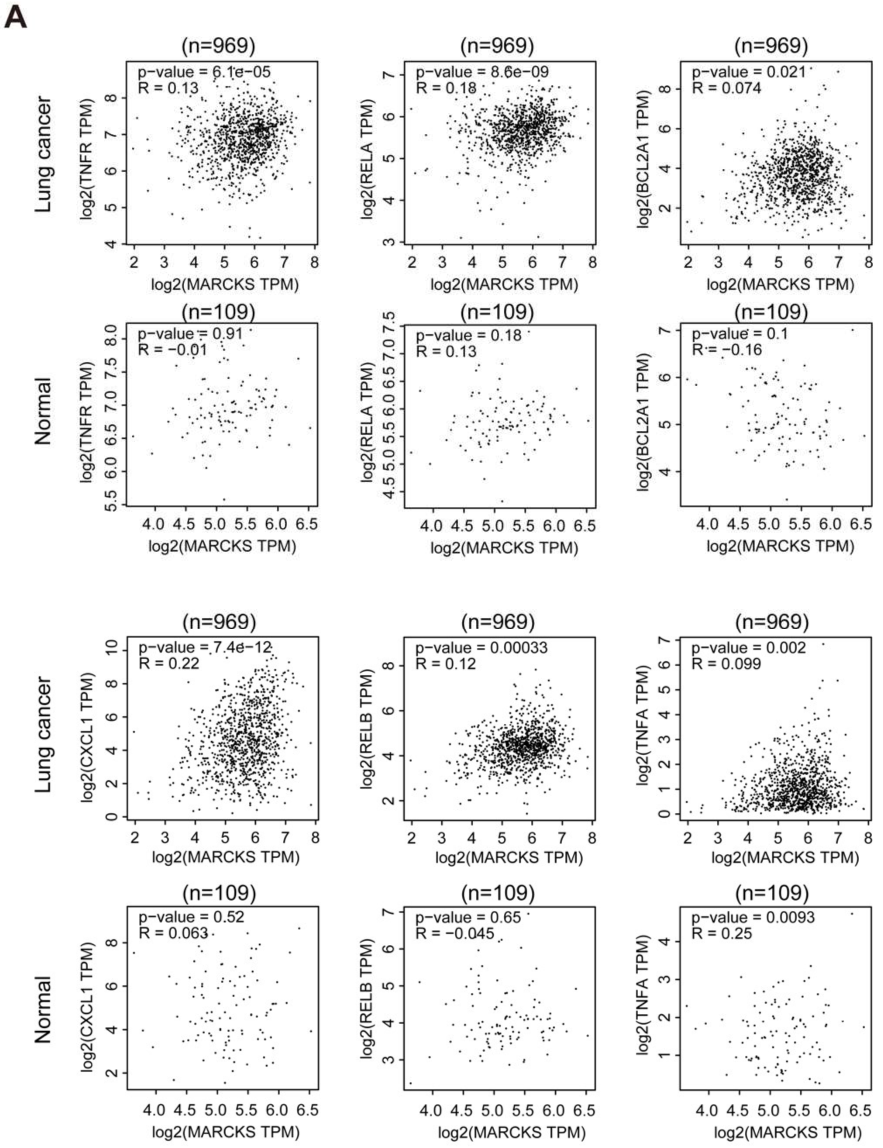

Supplementary Figure S4.

(**A**) Correlation analysis of MARCKS, TNFR, RELA, BCL2A1, CXCL1, RELB and TNFA genes in lung cancer samples (n=969) from the Cancer Genome Atlas (TCGA) and normal samples (n=109) from the Genotype-Tissue Expression (GTEx) databases by using a GEPIA online tool (http://gepia2.cancer-pku.cn/#index).

Supplementary Figure S5.

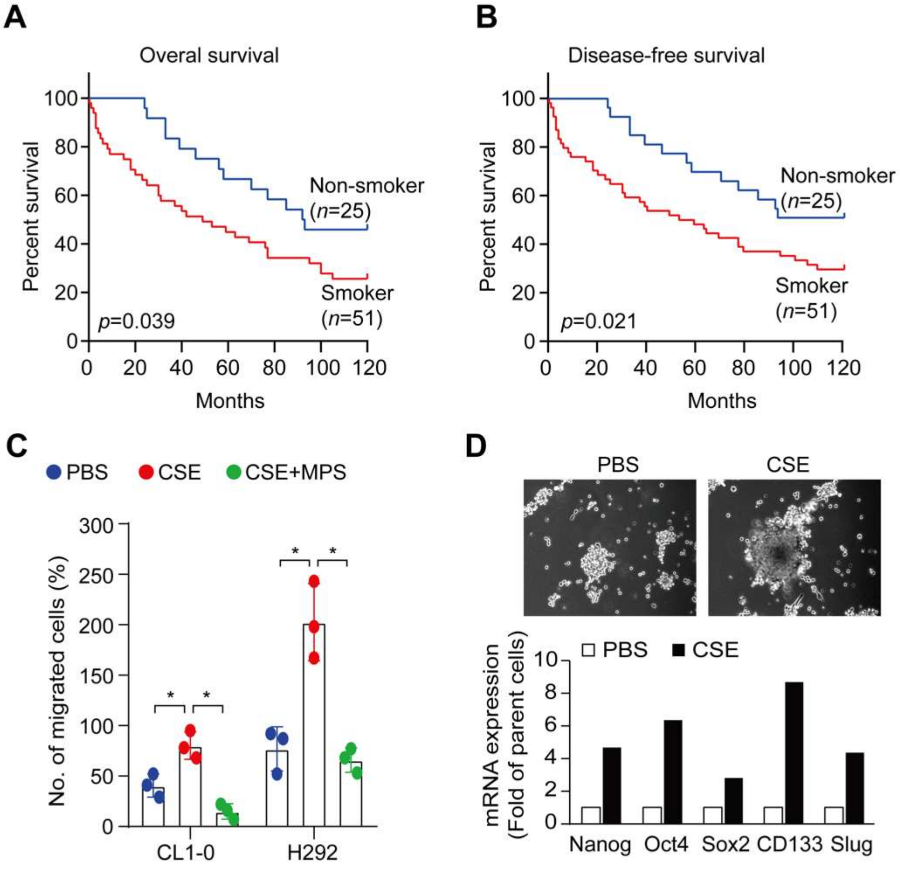

Supplementary Figure S5.

(**A-B**) The overall survival and disease-free survival of lung cancer patients from smokers (n=51) and non-smokers (n=25) was analyzed by the Kaplan–Meier and log-rank test. (**C**) Quantification of migrating ability of CL1-0 and H292 cells in response of CSE treatment in the presence or absence of 50 μM MPS using wound healing assays. Data from three independent experiments are represented as mean ± SD; *, $p < 0.05$. (**D**) Top, phase contrast photomicrographs of oncospheres in non-adherence 3-D culture without (left) and with 10% CSE (right). Bottom, RT-qPCR analyses of mRNA expression in the above cells.

Supplementary Figure S6.

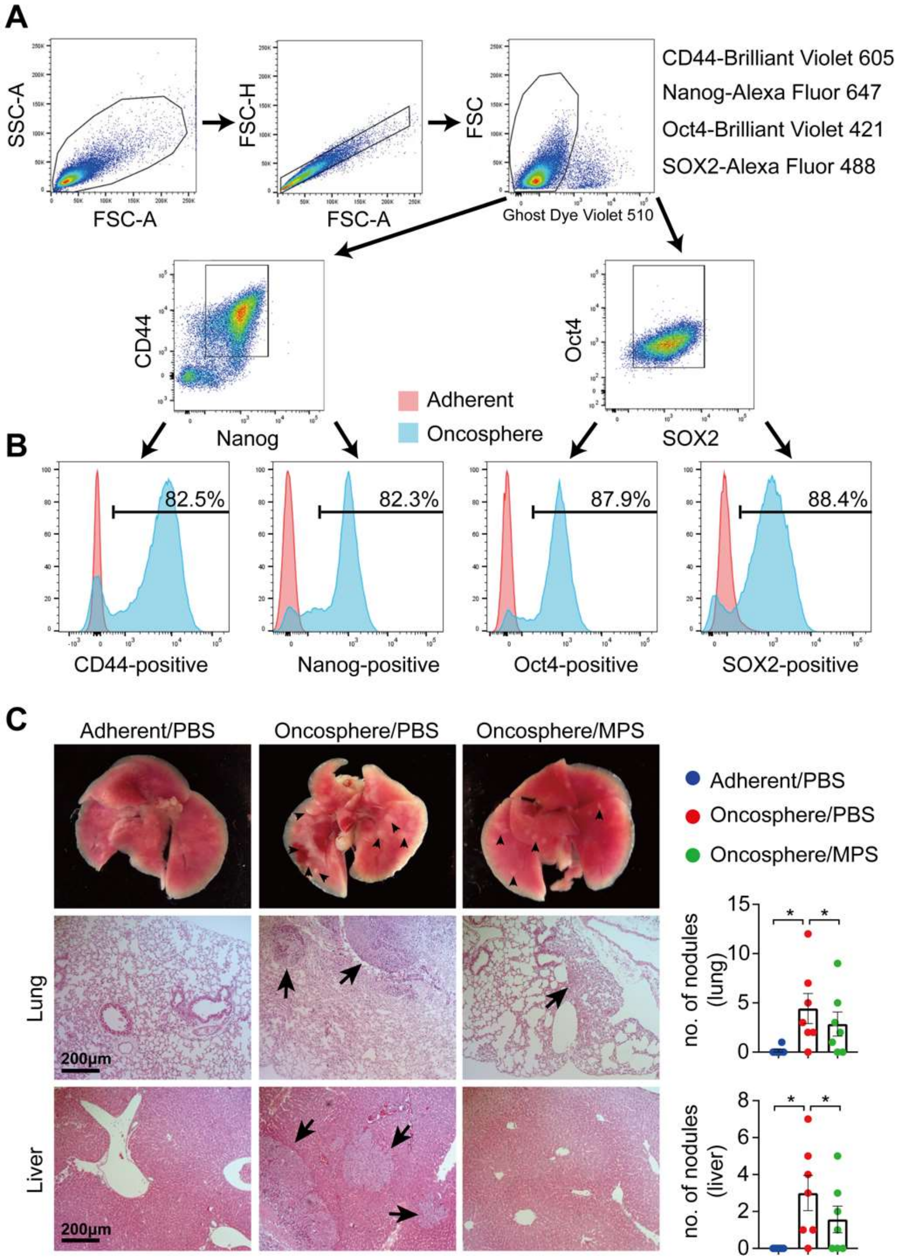

Supplementary Figure S6.

(**A, B**) Gating strategy (**A**) and quantification of flow cytometry analyses (**B**) for cancer stemness-associated markers in A549 cells derived from either adherent or oncosphere culture conditions. (**C**) Dissociated cells were intravenously injected into the lateral tail veins of six-week-old NOD SCID mice. After two weeks, mice were injected intraperitoneally with either PBS or PBS containing MPS peptide (28 mg/kg) once every two days for 21 days. At day 35, mice were sacrificed, and organs were removed and examined. Left, gross (top) and H&E staining (bottom) pictures of various organs removed from mice. The arrows indicate tumor nodules in the organ. Scale bar: 200 μm. Right, quantification of the average lung and liver metastasis nodules from mice injected with cancer cells and treated with PBS or MPS peptide as described. Data are represented as mean ± SEM (n=7); *, $p < 0.05$.

Supplementary References

1. Chen CH, Thai P, Yoneda K, Adler KB, Yang PC, Wu R. A peptide that inhibits function of Myristoylated Alanine-Rich C Kinase Substrate (MARCKS) reduces lung cancer metastasis. Oncogene. 2014; 33: 3696-706.
2. Thai P, Statt S, Chen CH, Liang E, Campbell C, Wu R. Characterization of a novel long noncoding RNA, SCAL1, induced by cigarette smoke and elevated in lung cancer cell lines. Am J Respir Cell Mol Biol. 2013; 49: 204-11.
3. Wang L, Joad JP, Zhong C, Pinkerton KE. Effects of environmental tobacco smoke exposure on pulmonary immune response in infant monkeys. J Allergy Clin Immunol. 2008; 122: 400-6, 6 e1-5.
4. Chen CH, Chiu CL, Adler KB, Wu R. A novel predictor of cancer malignancy: up-regulation of myristoylated alanine-rich C kinase substrate phosphorylation in lung cancer. Am J Respir Crit Care Med. 2014; 189: 1002-4.
5. Chen CH, Statt S, Chiu CL, Thai P, Arif M, Adler KB, et al. Targeting myristoylated alanine-rich C kinase substrate phosphorylation site domain in lung cancer. Mechanisms and therapeutic implications. American journal of respiratory and critical care medicine. 2014; 190: 1127-38.
6. Yang DC, Li JM, Xu J, Oldham J, Phan SH, Last JA, et al. Tackling MARCKS-PIP3 circuit attenuates fibroblast activation and fibrosis progression. FASEB J. 2019; 33: 14354-69.
7. Chen CH, Chuang SM, Yang MF, Liao JW, Yu SL, Chen JJ. A novel function of YWHAZ/beta-catenin axis in promoting epithelial-mesenchymal transition and lung cancer metastasis. Mol Cancer Res. 2012; 10: 1319-31.
